# Targeting osteoarthritis with small extracellular vesicle therapy: potential and perspectives

**DOI:** 10.3389/fbioe.2025.1570526

**Published:** 2025-06-20

**Authors:** Alba González-Rodríguez, F. Javier De Toro, Alberto Jorge-Mora, Pablo Fernandez-Pernas, Carlota Probaos Rivadulla, María Fraga, Juan A. Fafián-Labora, María C. Arufe

**Affiliations:** ^1^ Servicio de Cirugía Plástica, Complexo Hospitalario Universitario de A Coruña (CHUAC), A Coruña, Spain; ^2^ Grupo de Terapia Celular Medicina, Instituto de Investigación Biomédica de A Coruña (INIBIC), Complexo Hospitalario Universitario de A Coruña (CHUAC), Sergas/ Universidade da Coruña (UDC), As Xubías, A Coruña, Spain; ^3^ CICA - Centro Interdisciplinar de Química e Bioloxía e, Departamento de Bioloxía, Facultade de Ciencias, Universidade da Coruña (UDC), Campus de Elviña, CICA, A Coruña, Spain; ^4^ Departamento de Fisioterapia, Medicina y Ciencias Biomédicas, Facultad de Ciencias de la Salud, Universidade da Coruña (UDC), Campus de Elviña, CICA, A Coruña, Spain; ^5^ Cirugíía Ortopédica y Traumatología / Complejo Hospitalario de A Coruña, A Coruña, Spain; ^6^ Centre of Biological Sciences, University of Veterinary Medicine of Vienna, Vienna, Austria; ^7^ Department of Anatomical Pathology, University Hospital Complex A Coruña, A Coruña, Spain

**Keywords:** small extracelllular vesicles, osteoarthiritis, therapy, biomarker, personalized medicine

## Abstract

Osteoarthritis (OA) is a degenerative joint disease marked by inflammation, cartilage degradation, and pain, leading to a significant decline in quality of life. Recent advancements in extracellular vesicle (EV) research have introduced new therapeutic possibilities, with small extracellular vesicles (sEV) emerging as a promising strategy for OA treatment. sEV, particularly those derived from mesenchymal stem cells (MSCs), synoviocytes, chondrocytes, and induced pluripotent stem cells (iPSCs), demonstrate substantial anti-inflammatory and regenerative properties. These nanosized vesicles facilitate intercellular communication, delivering bioactive molecules that can modulate the joint microenvironment, promote chondrogenesis, and alleviate pain. Preclinical and early clinical studies indicate that sEV-based therapies may slow disease progression and enhance cartilage repair in OA patients. Despite the promising potential, challenges remain, including standardizing isolation techniques, understanding underlying mechanisms, and navigating regulatory pathways. This systematic review analyzes relevant publications published between 2019 and 2025, highlighting the therapeutic and biomarker potential of sEV in OA. Although there is substantial ongoing research into sEV and biomarkers, the fundamental understanding of OA pathogenesis remains largely unchanged, with most studies continuing to focus on established mechanisms of cartilage degradation, inflammation, and subchondral bone changes. The findings suggest that while therapeutic research into sEV is progressing, advancements in unraveling new pathophysiological mechanisms of OA are more limited. Further research is essential to optimize therapeutic protocols and establish clinical efficacy, marking sEV-based therapies as a promising but evolving approach for OA treatment.

## 1 Introduction

Osteoarthritis (OA) is one of the most common degenerative joint disorders and a major contributor to global, particularly among the aged population. It is a complex, multifactorial disease characterized by the progressive degeneration of articular cartilage, Changes in subchondral bone, formation of osteophytes, and inflammation of the synovial membrane (Xu et al., 2025). These alterations lead to chronic pain, joint stiffness, functional impairment, and a reduced quality of life. Despite the high prevalence and socioeconomic burden of OA, effective disease-modifying treatments remain elusive.

Currently, OA management relies heavily on conservative strategies such as non-steroidal anti-inflammatory drugs (NSAIDs), corticosteroid injections, physical therapy, and lifestyle modifications, with joint replacement surgery rather than targeting the underlying biological mechanisms driving cartilage degradation and joint dysfunction (Figueroa-Valdés et al., 2025). This therapeutic gap underscores the urgent need for innovative, disease-modifying interventions that can halt or even reverse OA progression.

Extracellular vesicles (EV) are membrane-bound particles released by cells that play essential roles in intercellular communication, particularly in the context of osteoarthritis (OA), where they influence inflammation, cartilage degradation, and tissue regeneration. EV are broadly categorized into small EVs (sEV), typically under 200 nm, encompassing exosomes (30–150 nm) that originate from the endosomal system and large EV (lEV), which include microvesicles (100–1,000 nm) shed directly from the plasma membrane, as well as apoptotic bodies (500–5,000 nm) released during programmed cell death. Various isolation techniques are employed to obtain EV from biological fluids or cell culture media, each with distinct benefits and limitations (Gupta et al., 2021). Differential ultracentrifugation is widely used to separate EVs based on size and density, while density gradient centrifugation improves purity. Size exclusion chromatography allows for the separation of EVs from soluble proteins and smaller particles, and polymer-based precipitation (e.g., with polyethylene glycol) offers a rapid, scalable option (Yuan et al., 2024). More advanced methods, such as immunoaffinity capture and microfluidic platforms, enable greater specificity and are increasingly adopted for isolating defined EV subpopulations, crucial for investigating their diverse roles in OA pathology and therapeutic potential.

In recent years, regenerative medicine has emerged as a promising avenue for OA treatment. Among the various regenerative strategies under investigation, sEV have gained significant attention due to their intrinsic capacity for intercellular communication and their ability to modulate a variety of biological processes. sEV are nanoscale membrane-bound vesicles released by almost all cell types and contain a rich cargo of proteins (Ruiz et al., 2020; Zhang et al., 2021; Li et al., 2022; Nguyen et al., 2022; Ragni et al., 2022b; Genestra et al., 2023; Cheng et al., 2024; Forteza-Genestra et al., 2024; Mohd Noor et al., 2024; Scalzone et al., 2024; Wu R. et al., 2024), lipids (Kolhe et al., 2020; Mustonen et al., 2022; Zhang et al., 2023), mRNAs (Li et al., 2024c), and microRNAs (LIN et al., 2021). These vesicles play critical roles in maintaining tissue homeostasis and orchestrating cellular responses in both physiological and pathological conditions.

Preclinical studies have demonstrated that sEV derived from mesenchymal stem cells (MSCs), chondrocytes, and synoviocytes exert anti-inflammatory, anti-apoptotic, and pro-regenerative effects in the context of OA. For instance, MSC-derived sEV have been shown to promote cartilage repair, modulate immune responses, and inhibit matrix-degrading enzymes (Tang et al., 2021; Morente-López et al., 2022; Warmink et al., 2023). Likewise, chondrocyte-derived sEV can enhance extracellular matrix synthesis (Shang et al., 2021; Hosseinzadeh et al., 2023), while synoviocyte-derived sEV may influence synovial homeostasis and reduce joint inflammation (Chen et al., 2022b; Asghar et al., 2024). These findings suggest that sEV-based therapies hold substantial potential as minimally invasive, cell-free approaches for OA treatment.

However, despite this promising landscape, several critical gaps in the literature remain. Firstly, the precise molecular mechanisms through which sEV exert their therapeutic effects in OA are not yet fully elucidated, with much of the existing evidence limited to *in vitro* or small-animal models. Secondly, there is a lack of standardized protocols for the isolation, purification, and characterization of sEV, leading to variability in experimental outcomes and complicating cross-study comparisons. Thirdly, the comparative efficacy of sEV derived from different cell sources remains poorly defined, as does the optimal route of administration, dosage, and treatment frequency. Moreover, while some early-phase clinical trials are underway, robust clinical evidence supporting the safety, efficacy, and long-term outcomes of sEV-based therapies in human OA patients is still lacking.

This review seeks to address these deficiencies by providing a comprehensive and critical analysis of current research on the application of sEV in OA therapy. Specifically, it aims to 1) examine the therapeutic mechanisms of sEV in cartilage regeneration, immune modulation, and inflammation resolution; 2) compare the regenerative potential of sEV derived from various cell sources; and 3) discuss the key translational challenges and considerations for clinical application, including regulatory hurdles, manufacturing scalability, and safety concerns.

By integrating and synthesizing the latest findings in this rapidly evolving field, this manuscript contributes to a more nuanced understanding of sEV as emerging therapeutic agents for OA and highlights avenues for future research and clinical translation. Addressing these critical questions is essential for advancing sEV-based therapies from experimental models toward safe and effective clinical applications that could transform the management of osteoarthritis.

## 2 Methodology

### 2.1 Search strategy

A systematic literature search was conducted using the PubMed and Web of Science databases. The search strategy combined keywords and Medical Subject Headings (MeSH) relevant to *osteoarthritis* and *small extracellular vesicles (sEV)*. Boolean operators (AND, OR) were used to refine search results.

Search terms included: “osteoarthritis,” “small extracellular vesicles,” “exosomes,” “extracellular vesicles,” “cartilage breakdown,” “matrix metalloproteinases (MMPs),” “aggrecanases,” “subchondral bone changes,” “sclerosis formation,” “osteophyte formation,” “synovial inflammation,” and “pro-inflammatory cytokines.”

The search was limited to articles published between January 2019 and March 2025.

### 2.2 Eligibility criteria

Studies were selected using the PICO framework:

• Population: Human patients, animal models, or *in vitro* systems affected by osteoarthritis.
• Intervention: Administration or analysis of sEV.
• Comparator(s): Placebo, untreated controls, or standard interventions (if reported).
• Outcomes: Biomarker changes and therapeutic efficacy in OA models.

Inclusion Criteria:

• Original research on the role of sEV in OA pathophysiology, diagnosis, or treatment.
• Articles published in English in peer-reviewed journals.
• Studies employing human, animal, or *in vitro* models.

Exclusion Criteria:

• Review articles, meta-analyses, editorials, or abstracts without original data.
• Studies not directly related to OA or sEV
• Articles focusing solely on large extracellular vesicles or unrelated vesicles subtypes.
• Non-English publications.

### 2.3 Data sources and study selection

Two independent reviewers (Reviewer A and Reviewer B) screened all retrieved titles and abstracts for relevance. Full-text articles were then assessed based on the predefined inclusion and exclusion criteria. Disagreements were resolved through discussion or by a third reviewer (Reviewer C). No automation tools were used in this stage.

A total of 162 studies were ultimately included in the qualitative synthesis. These were grouped into three main thematic areas based on the primary focus of each publication:

39 studies focused on OA pathogenesis, including cartilage degradation, synovial inflammation, and subchondral bone changes.

122 studies investigated the therapeutic potential of sEV in OA, primarily using *in vitro* or *in vivo* preclinical models.

22 studies evaluated sEV as potential biomarkers for OA diagnosis, prognosis, or treatment response.

### 2.4 Data extraction

Data from the included studies were extracted using a standardized data extraction form by two reviewers independently. Information collected included:

• Study characteristics (author, publication year, journal, study design)
• Type of model used (*in vivo* or *in vitro*)
• Outcomes measured, including:

Biomarkers in OA: inflammatory cytokines (IL-1β, TNF-α), matrix metalloproteinases (MMP-13), aggrecanases, cartilage-specific proteins (collagen II, aggrecan); measured by ELISA, RT-PCR, or immunohistochemistry.

Therapeutic efficacy: improvements in joint structure or function, evaluated via WOMAC scores, MRI/CT imaging, or histological scoring systems.

All extracted data were cross-verified, and disagreements were resolved by consensus. No data automation tools were employed, and no study authors were contacted for additional data.

• Key findings and conclusions.

### 2.5 Quality assessment and risk of bias

Quality and risk of bias were assessed independently by two reviewers using appropriate tools:

• For *in vivo* studies: ARRIVE (Animal Research: Reporting of *in vivo* Experiments) guidelines.
• For *in vitro* studies: Internal quality assessment criteria were developed based on reproducibility, experimental controls, and reporting clarity.
• For all studies: Risk of bias was evaluated using a modified version of the SYRCLE Risk of Bias tool for preclinical studies, and criteria included randomization, blinding, and outcome reporting.

Each study was assessed by two reviewers working independently. Discrepancies were resolved via discussion or consultation with a third reviewer.

### 2.6 Data synthesis

All included studies were synthesized qualitatively. Narrative summaries were used to describe key findings and thematic patterns. Where appropriate, data were tabulated to compare models, outcomes, and results across studies.

The 39 studies related to OA pathogenesis were further divided into three subcategories: Cartilage Breakdown (n = 23); synovial Inflammation (n = 7); subchondral bone changes (n = 9).

The 122 therapeutic studies assessed outcomes such as cartilage repair, inflammation reduction, and functional improvements using sEV derived from various cell sources, most commonly MSCs. The 22 biomarker studies explored the diagnostic potential of sEV cargo.

No meta-analysis was conducted due to heterogeneity in study designs and outcome measures.

### 2.7 Ethical considerations

This systematic review adhered to the PRISMA (Preferred Reporting Items for Systematic Reviews and Meta-Analyses) guidelines. As the review did not involve primary data collection, ethical approval was not required.

## 3 Results

**FIGURE 1:**
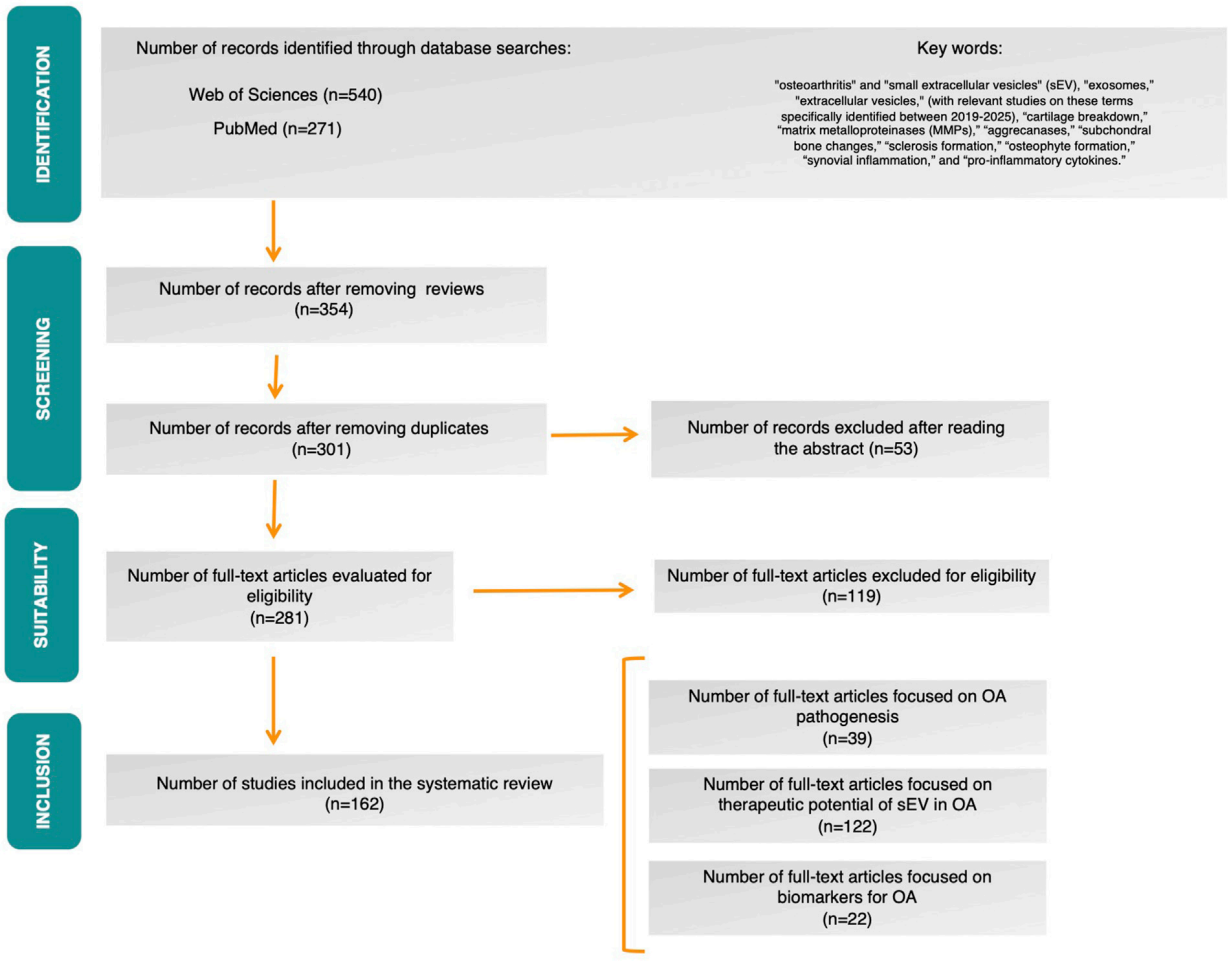
PRISMA flow diagram.

A total of 354 records were retrieved from the Web of Science and PubMed databases after duplicate removal. After screening the titles and abstracts, 53 records were excluded, leaving 301 articles to be assessed after reading the abstract. Following full-text screening, 281 scientific papers meeting these criteria were selected. After excluding studies that did not meet the specific selection criteria, *in vitro*, *in vivo* and clinical studies, out of a total of 162 scientific papers, were included in the qualitative synthesis. The PRISMA flow diagram is shown in Figure 1.

In our systematic review, we analyzed a total of 39 relevant publications published between 2019 and 2025, focusing on key aspects of osteoarthritis (OA) pathogenesis. However, to provide a comprehensive state of the art and due to the lack of significant advances in recent years, we also included studies prior to 2019, as earlier works remain crucial for understanding the fundamental basis of the disease. These publications were grouped into three main categories:

i) Cartilage Breakdown: 23 publications discussed cartilage degradation mediated by factors such as MMPs and aggrecanases.
ii) Synovial Inflammation: 7 publications addressed the role of pro-inflammatory cytokines, such as IL-1β and TNF-α, in synovial inflammation.
iii) Subchondral Bone Changes: 9 publications discussed changes in the subchondral bone, including sclerosis and osteophyte formation.

**FIGURE 2:**
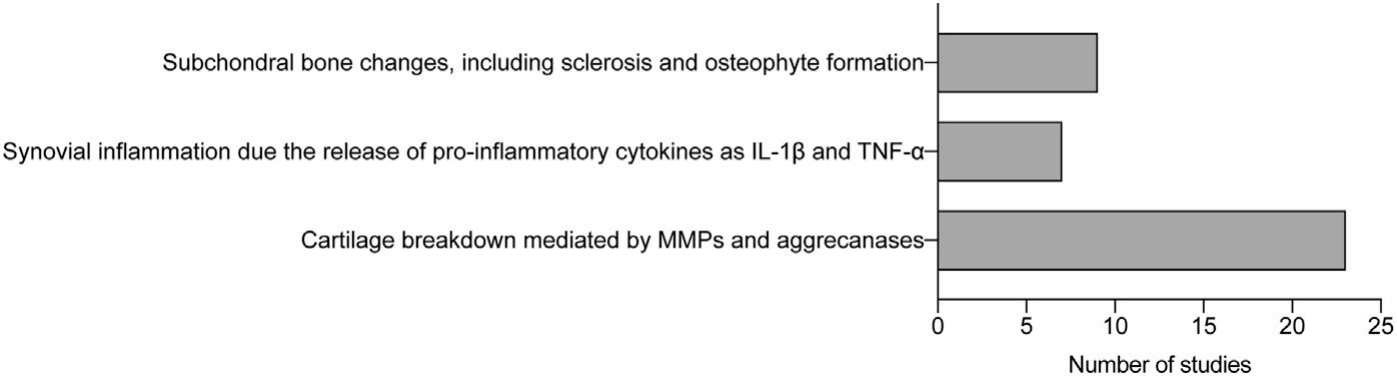
Analysis of studies about osteoarthritis pathogenesis. Histogram of number of studies classified by key pathological features. IL-1β (Interleukin-1β); TNF-α (Tumor Necrosis Factor-α); MMPs (matrix metalloproteinases).

The inclusion of studies prior to 2019 highlights that, the foundational knowledge of OA pathogenesis remains relatively stable. Most of the relevant studies build upon established concepts such as cartilage degradation mechanisms and inflammatory processes, which have been extensively studied in previous years. This suggests that while therapeutic research is advancing, progress in uncovering new aspects of OA pathogenesis has been limited. The current focus appears to be more on applying existing knowledge to explore new therapeutic avenues rather than discovering new pathophysiological mechanisms (Figure 2).

In addition to the pathogenic mechanisms, 122 studies investigated the therapeutic potential of sEV in OA, primarily in preclinical models. These studies highlighted the regenerative, anti-inflammatory, and chondroprotective effects of sEV derived from sources such as mesenchymal stem cells (Kouroupis et al., 2023; Warmink et al., 2023), chondrocytes (Wang et al., 2020; Chen et al., 2024; Feng et al., 2024a; Yan et al., 2024), and synoviocytes (Lu et al., 2021; Lai et al., 2023; Wang and Sun, 2023; Cao H. et al., 2024). Furthermore, 22 publications evaluated EVs as potential biomarkers for OA diagnosis, progression monitoring, and treatment response prediction. Together, these findings underscore the growing interest in EVs as both mechanistic contributors to OA pathology and promising tools for therapy and clinical translation.

**FIGURE 3:**
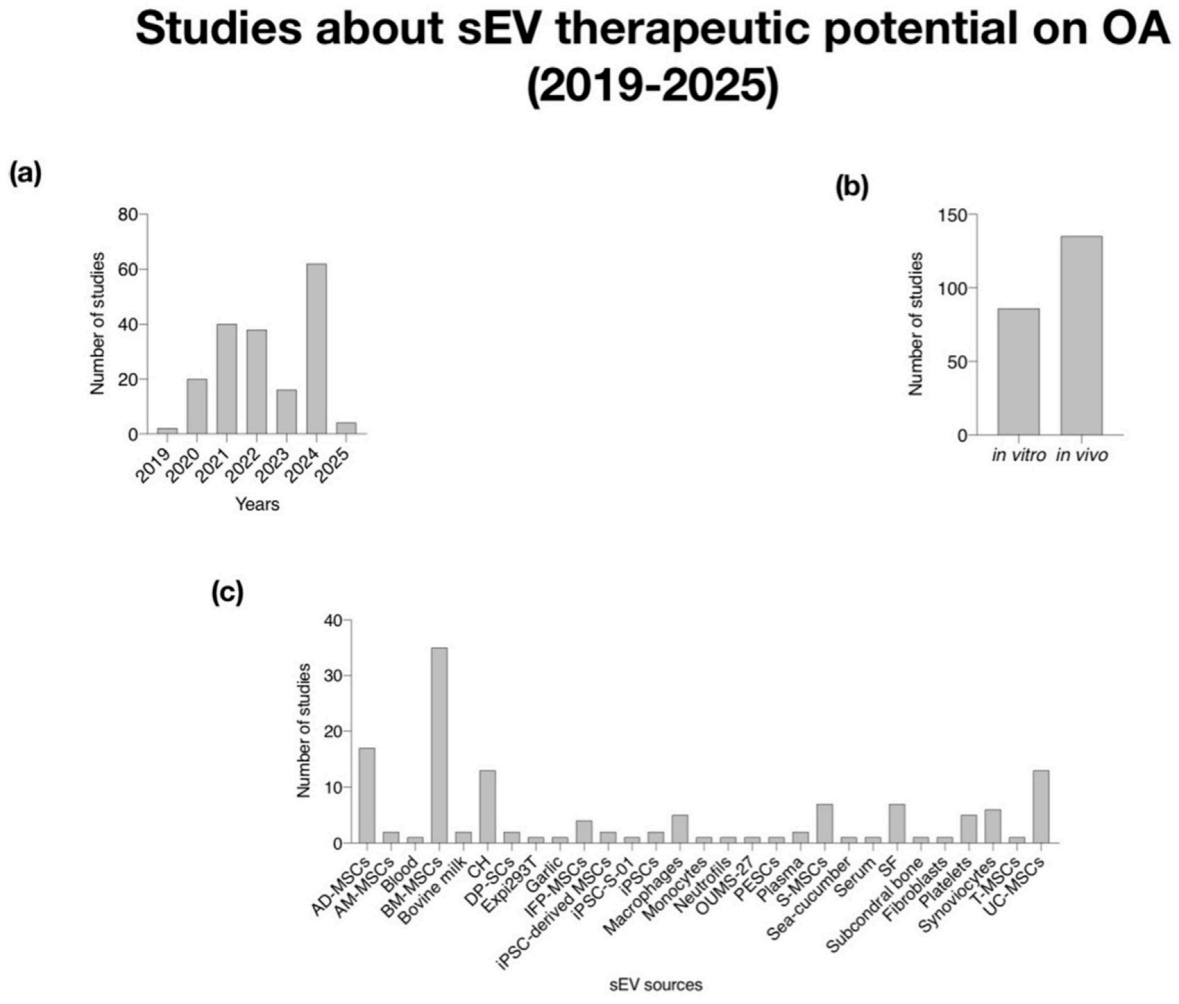
Analysis of studied about sEV therapeutic potential on OA (2019–2025). **(a)** Histogram of number of studies per year; **(b)** Histogram of number of studies *in vitro* and *in vivo*; **(c)** Histogram of the number of studies classified by sEV sources. AM-MSCs (Mesenchymal Stem Cells from Ammiotic Membrane); AD-MSCs (Mesenchymal Stem Cells from Adipose Tissue); BM-MSCs (Mesenchymal Stem Cells from Bone Marrow); DP-SCs (Dental Pulp-derived Stem Cells); IFP-MSCs (Infrapatellar Fat Pad-derived Mesenchymal Stem Cells); iPSC-derived MSCs (Mesenchymal Stem Cell-derived iPSCs); iPSCs (induced Pluripotent Stem Cells); S-MSCs (Mesenchymal Stem Cells from synovial membrane); T-MSCs (Tonsil-derived Mesenchymal Stem Cells); SF (Synovial Fluid); UC-MSCs (Umbilical Cord-derived Mesenchymal Stem Cells).

**FIGURE 4:**
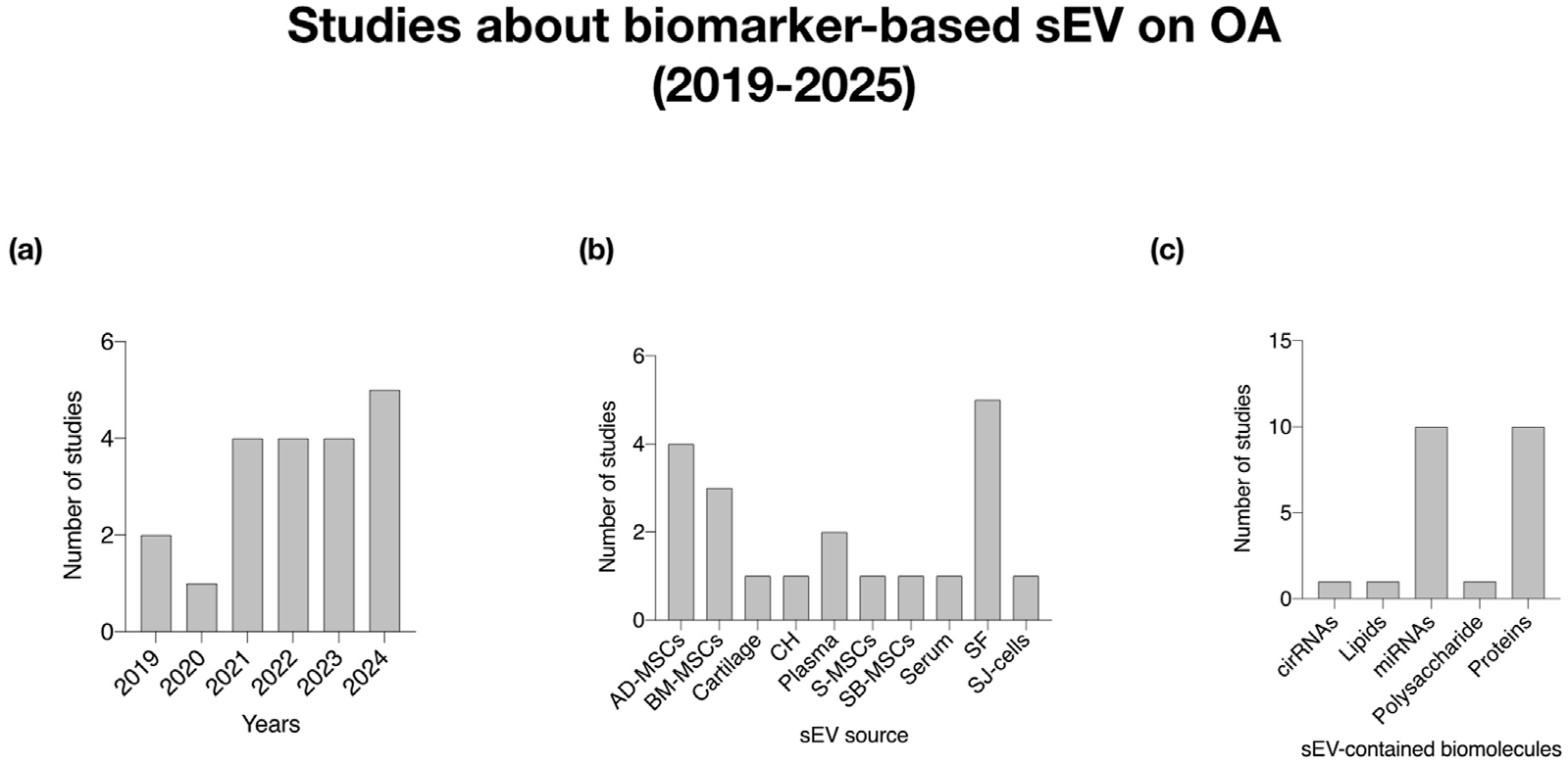
Analysis of studies about sEV cargo as biomarker in OA (2019–2025). **(a)** Histogram of number of studies per year; **(b)** Histogram of the number of studies classified by sEV sources; **(c)** Histogram of number of studies based on sEV-contained biomolecules. CH (Chondrocytes); SJ-cells (Cells from Synovial Joint); AD-MSCs (Mesenchymal Stem Cells from Adipose Tissue); BM-MSCs (Mesenchymal Stem Cells from Bone Marrow).

The consistent upward trend in the number of studies from 2019 to 2025 indicates a growing recognition of sEV as a significant focus in OA research. This growth reflects an expanding understanding of their therapeutic potential and biomarker capabilities in managing OA. A notable increase in publications in 2024 specifically highlights the therapeutic potential and biomarker applications of mesenchymal stem cells (MSCs) and synovial fluid in OA research (Figures 3a, 4a).

The rise in studies, particularly in 2024, points to an expanding interest in the therapeutic potential of sEV derived from MSCs and their roles as biomarkers in OA research. This reflects a deeper understanding of how sEV can be leveraged for both treatment and diagnostic purposes.

The distribution of studies between *in vitro* and *in vivo* research suggests a systematic approach to validating the effects of sEV before pre-clinical application (Figure 3b). The balance between these research approaches indicates thorough validation, with *in vitro* studies providing foundational insights on mechanisms, while *in vivo* studies contribute to understanding the efficacy and safety of sEV therapies in real biological contexts.

The variety of sEV sources identified, including various types of MSCs, plasma, serum, synovial fluid, and others, demonstrates the multifaceted nature of sEV research. The increasing research on MSC-derived sEV, including adipose-derived and bone marrow, highlights their diverse therapeutic potential. Additionally, studies on sEV from synovial fluid emphasize their role in OA treatment, reinforcing the unique properties derived from multiple sources. The inclusion of additional sources such as macrophages and platelets suggest a comprehensive exploration of immune and cellular responses tied to OA pathology (Figure 3c, 4b).

The analysis of sEV-contained biomolecules like miRNAs (10 publications), proteins (9 publications), circRNAs (1 publication), and lipids (1 publication) underscores the relevance of these molecules as potential biomarkers. Notably, miRNAs and proteins emerge as the most extensively studied and utilized biomolecules in sEV research. This highlights the dual role of sEV as both therapeutic agents and diagnostic tools. An increased examination of these biomolecules, particularly miRNAs and proteins from MSCs (10 publications) and synovial fluid (5 publications), suggests that specific sEV-associated biomarkers may lead to personalized treatment strategies (Figure 4c).

The trends observed in both therapeutic and biomarker studies highlight critical avenues for future research, including the need for standardized methodologies and larger clinical trials. The significant volume of research, particularly emphasizing MSCs and synovial fluid, highlights the necessity of refining clinical applications of sEV in OA management. These endeavors could enhance the legitimacy and applicability of sEV therapies in clinical practice for OA.

The insights from this systematic review integrate the intricate relationship between the growing body of literature on sEV, their diverse sources, and their potential roles as both therapeutic tools and biomarkers in OA. This interconnected understanding fosters a holistic approach to addressing OA, paving the way for innovative research and potential clinical advancements in the coming years. The research underscores the interconnectedness of sEV, their diverse sources, and their roles in OA management, supporting a comprehensive approach to addressing this condition while encouraging innovative research and clinical advancements in the field.

This systematic review clarifies the potential impact of sEV, especially those derived from MSCs and synovial fluid, in OA. It also sets the foundation for identifying critical areas for future exploration, particularly given the increase in studies focusing on these topics in 2024.

## 4 Discussion

### 4.1 Pathophysiology of OA

OA is a multifactorial disease driven by mechanical stress, inflammation, and metabolic changes. Key pathological features include:

#### 4.1.1 Cartilage breakdown mediated by matrix metalloproteinases (MMPs) and aggrecanases

Cartilage breakdown is a hallmark of OA and other joint degenerative conditions. It is primarily mediated by two enzymatic families: matrix metalloproteinases (MMPs) and aggrecanases. These enzymes degrade the extracellular matrix (ECM), which is crucial for maintaining cartilage integrity and function. While MMPs and aggrecanases target different ECM components, their activities are interrelated. For instance, MMPs can degrade the non-collagenous domains of aggrecan, complementing aggrecanase-mediated cleavage (Madsen et al., 2010). Moreover, MMP activity is essential for aggrecanase activation under certain pathological conditions (Clements et al., 2011). Below, we synthesize findings from key studies to elucidate the roles of MMPs and aggrecanases in cartilage degradation. MMPs are a family of zinc-dependent endopeptidases that degrade various ECM components. Among these, MMP-3 (stromelysin-1) and MMP-13 (collagenase-3) are particularly implicated in cartilage breakdown. MMP-13 degrades type II collagen, a critical structural component of cartilage, while MMP-3 activates other MMPs and contributes to proteoglycan loss (Cawston et al., 1998; Stanton et al., 2002). Elevated MMP activity is observed in inflammatory and degenerative joint diseases, where cytokines such as IL-1β and TNF-α drive their expression (Tsushima et al., 2012).

MMP-mediated cartilage degradation is tightly regulated by tissue inhibitors of metalloproteinases (TIMPs). TIMP-3 (tissue inhibitor of metalloproteinase-3) is particularly significant, as it inhibits both MMPs and aggrecanases, thereby preserving cartilage ECM integrity (Gendron et al., 2003; Li et al., 2004). However, dysregulation of the TIMP/MMP balance contributes to cartilage destruction in OA. MMPs, particularly MMP-3 and MMP-13, degrade cartilage collagens and proteoglycans, while aggrecanases (ADAMTS-4, ADAMTS-5) target aggrecan, a major cartilage proteoglycan, leading cartilage breakdown (Fosang et al., 2000; Stanton et al., 2002; Madsen et al., 2010). Pro-inflammatory cytokines like IL-1β and IL-17 upregulate MMP and aggrecanase expression, intensifying cartilage degradation. IL-17 also stimulates nitric oxide production, suppressing matrix synthesis (Cai et al., 2001; 2002; Huang et al., 2020). TIMP-3 effectively inhibits aggrecanases, while selective metalloproteinase inhibitors reduce cartilage degradation (Gendron et al., 2003).

Aggrecanases, primarily ADAMTS-4 and ADAMTS-5 (a disintegrin and metalloproteinase with thrombospondin motifs), are specialized for degrading aggrecan, the predominant proteoglycan in cartilage. Aggrecan degradation compromises cartilage’s load-bearing properties, exacerbating joint dysfunction (Fosang et al., 2000; Madsen et al., 2010). Aggrecanase activity is often upregulated in response to pro-inflammatory mediators, such as IL-1 and IL-17 (Cai et al., 2001; Wilkinson et al., 2017). Studies have highlighted the molecular characteristics of aggrecan fragments generated during degradation. These fragments serve as biomarkers for cartilage turnover and disease progression (Patel et al., 2007). Importantly, targeted inhibition of aggrecanases has shown promise in preclinical models, reducing cartilage degradation, and improving joint function (Kanakis et al., 2019).

While MMPs and aggrecanases target different ECM components, their activities are interrelated. For instance, MMPs can degrade the non-collagenous domains of aggrecan, complementing aggrecanase-mediated cleavage (Madsen et al., 2010). Moreover, MMP activity is essential for aggrecanase activation under certain pathological conditions (Clements et al., 2011).Nutraceuticals like luteolin and crocin attenuate MMP and aggrecanase activity (Gendron et al., 2003; Moncada-Pazos et al., 2011). Mechanical stress, injury, and oxidative stress induce MMP/aggrecanase expression. Pathways such as ERK, NF-κB, and PKC play critical roles in this regulation (Maeda et al., 2001; Haslauer et al., 2013). Targeted aggrecanase inhibition (e.g., ADAMTS-5) reduces cartilage damage and preserves joint function. Experimental drugs like suramin and recombinant proteins (e.g., TSG-6) show promise in modulating MMP/aggrecanase activity (Kanakis et al., 2019; Drummond et al., 2023).

Cartilage degradation involves both reversible and irreversible processes, depending on the enzymes involved. This study showed that aggrecanase-mediated degradation, especially in early stages, can be reversed with anabolic stimuli like IGF-1, leading to restored proteoglycan content and ECM integrity. In contrast, once MMPs degrade aggrecan and type II collagen, particularly during later stages, the chondrocytes exhibit limited repair capacity. Collagen II formation was only significantly recovered at early time points, suggesting a narrow therapeutic window. Histological analysis confirmed glycosaminoglycan replenishment when intervention occurred early. These findings emphasize the timing and nature of enzymatic activity in OA progression. Therapies aimed at early aggrecanase inhibition may preserve regenerative potential. Meanwhile, MMP-targeted strategies are crucial for halting irreversible damage. The distinction between reversible and irreversible cartilage damage provides a framework for stage-specific OA treatment design (Karsdal et al., 2008). Elevated levels of degradation products (e.g., aggrecan fragments) and inflammatory markers (e.g., S100A8, Tenascin-C) serve as indicators of cartilage breakdown (Van Lent et al., 2008; Sofat et al., 2012). Hyaluronic acid (HA), for example, has been shown to partially inhibit MMP and aggrecanase activity, thereby protecting cartilage (Mladenovic et al., 2014). Similarly, small-molecule inhibitors, such as luteolin and mannosamine, selectively reduce aggrecanase activity, preserving ECM integrity (Patwari et al., 2000; Moncada-Pazos et al., 2011). Modulating the activity of MMPs and aggrecanases represents a therapeutic avenue for preventing cartilage breakdown. Emerging therapies also focus on enhancing endogenous protective mechanisms. For instance, microRNA-17 maintains cartilage homeostasis by suppressing MMP and aggrecanase expression (Zhang et al., 2022). Furthermore, TIMP-3 overexpression and recombinant Link protein supplementation have shown potential in reducing ECM degradation in experimental models (Drummond et al., 2023).

MMPs and aggrecanases are central to the pathogenesis of cartilage degradation, driven by inflammatory and mechanical stressors. Their interplay underscores the complexity of ECM turnover and highlights opportunities for therapeutic intervention. Future research should aim to refine selective inhibitors and explore regenerative strategies to restore cartilage homeostasis.

#### 4.1.2 Synovial inflammation due to the release of pro-inflammatory cytokines such as IL-1β and TNF-α

The release of pro-inflammatory cytokines, particularly IL-1β and TNF-α, plays a pivotal role in initiating and sustaining the inflammatory milieu. These cytokines trigger a cascade of molecular and cellular events that lead to synovial hyperplasia, fibroblast activation, cartilage degradation, and recruitment of immune cells. The interaction between pro-inflammatory cytokines and synovial fibroblasts plays a critical role in the pathogenesis of OA. Farina et al. demonstrated that the activation of synovial fibroblasts by IL-1β and TNF-α is further enhanced by the pro-β-nerve growth factor (proNGF) and its receptor p75NTR (Farina et al., 2022). This novel mechanism amplifies the inflammatory response, making it a promising therapeutic target. Additionally, synovial fibroblasts stimulated with these cytokines release mediators such as Trx80, a truncated form of thioredoxin. According to Lemarechal et al., Trx80 contributes to the perpetuation of inflammation by promoting further cytokine release and oxidative stress, exacerbating joint damage (Lemarechal et al., 2007). The synovial membrane plays a pivotal role in OA progression by responding to mechanical stress and wear particles. Harvanova et al. highlighted how *in vitro* models of OA can replicate these processes, demonstrating the synovial membrane’s contribution to cartilage and joint degradation (Harvanova et al., 2022). Park et al. further corroborated this, showing that polyethylene wear particles activate synovial inflammation, leading to cartilage damage and meniscus degradation (Park et al., 2013). The pro-inflammatory effects of cytokines extend beyond fibroblast activation. Filková et al. reported that interleukin-35 (IL-35), typically considered anti-inflammatory, can paradoxically exhibit pro-inflammatory effects in rheumatic arthritis (RA) (Filková et al., 2015). This dichotomy underscores the complex cytokine signaling in synovitis. Furthermore, oncostatin M (OSM), another cytokine involved in synovial inflammation, induces pro-inflammatory mediators via the JAK/STAT pathway. Weston et al. demonstrated that JAK inhibitors like baricitinib effectively block this pathway, reducing inflammation in synovial-derived cells. This highlights the therapeutic potential of targeted cytokine pathway inhibition. Targeted drug delivery systems have emerged as promising strategies for treating synovial inflammation (Weston et al., 2022). Hu et al. explored pH-responsive polymeric nanoparticles loaded with rhein, which selectively release the drug in the acidic inflammatory microenvironment. This innovation minimizes systemic side effects while directly addressing synovial inflammation.

Pro-inflammatory cytokines IL-1β and TNF-α drive synovial inflammation through multiple mechanisms, including fibroblast activation, cytokine crosstalk, and oxidative stress. Advances in understanding these pathways have unveiled novel therapeutic targets such as proNGF-p75NTR signaling, IL-35 modulation, and JAK/STAT inhibition. Additionally, innovative delivery systems like pH-sensitive nanoparticles present promising strategies to control inflammation locally and effectively (Hu et al., 2020).

Further studies integrating molecular, cellular, and translational approaches are essential for the development of targeted therapies to treat synovial inflammation and its sequelae in OA.

#### 4.1.3 Subchondral bone changes, including sclerosis and osteophyte formation

Changes in subchondral bone, including sclerosis and osteophyte formation, are key features that contribute to joint degeneration. Emerging studies provide detailed insights into the molecular and structural alterations underlying these changes. Subchondral bone sclerosis is a hallmark of OA, characterized by increased bone density and altered architecture. The thickened subchondral plate and trabecular remodeling are thought to result from mechanical stress and aberrant signaling pathways. Ruan et al. highlighted the multifaceted role of ALK3 signaling in OA development, demonstrating its involvement in subchondral bone sclerosis through the regulation of osteoblast activity and bone matrix production (Ruan et al., 2023). The study emphasizes the interplay between cartilage degradation and subchondral bone remodeling, wherein mechanical stress exacerbates both processes. Experimental models, such as the anterior cruciate ligament transection (ACLT) and meniscectomy in rats, have been instrumental in elucidating the mechanisms underlying sclerosis. Pickarski et al. observed significant thickening of the subchondral plate and increased mineralization in these models, accompanied by elevated levels of osteoclast and osteoblast activity markers (Pickarski et al., 2011). Automated micro-CT analysis further supports these findings, as Das Neves Borges et al. demonstrated precise quantification of bone volume and density changes in murine OA models, confirming the progressive nature of subchondral bone sclerosis (Das Neves Borges et al., 2017). Osteophytes, or bone spurs, are another hallmark of OA and represent an adaptive response to joint instability and cartilage damage. Their formation is driven by endochondral ossification and influenced by mechanical stress, inflammation, and signaling pathways. Liao et al. noted that osteophyte development in axial spondyloarthritis shares similarities with OA, particularly in the activation of growth factors like transforming growth factor-β (TGF-β), which stimulates mesenchymal stem cell differentiation into chondrocytes and osteoblasts (Liao et al., 2024). Wang et al. demonstrated the sequential progression of cartilage degradation, bone resorption, and osteophyte formation in a temporomandibular joint OA model (Wang et al., 2012). The study revealed that osteophytes arise in response to altered joint biomechanics and increased expression of pro-osteogenic factors. Additionally, Jimenez et al. observed spontaneous osteophyte formation in Dunkin Hartley guinea pigs, further corroborating the link between mechanical loading and osteophyte development. Increased physical activity, particularly under conditions of compromised cartilage integrity, accelerates osteophyte formation (Jimenez et al., 1997). Siebelt et al. reported severe osteophyte growth in pain-induced OA models subjected to excessive loading. These findings underscore the dual role of mechanical stress in promoting both sclerosis and osteophyte formation, highlighting the importance of joint biomechanics in OA pathophysiology. The interplay between cartilage and subchondral bone is critical in OA progression (Siebelt et al., 2014). Acute synovitis, as described by Liao et al., precedes subchondral bone changes and amplifies joint degeneration through inflammatory mediators that impact both tissues (Liao et al., 2020). Furthermore, Pickarski et al. and Hayami et al. identified coordinated molecular changes in articular cartilage and subchondral bone, suggesting a dynamic feedback loop that perpetuates joint damage. Sclerotic changes are not limited to structural alterations but also involve molecular shifts. Hayami et al. identified upregulation of bone morphogenetic proteins (BMPs) and Wnt signaling in subchondral bone during OA progression, further contributing to increased bone formation and sclerosis (Hayami et al., 2006; Pickarski et al., 2011).

Subchondral bone changes, including sclerosis and osteophyte formation, are integral to OA pathogenesis and progression. Advances in imaging and molecular analysis have deepened our understanding of these processes, offering potential therapeutic targets. Interventions aimed at modulating mechanical stress, inflammation, and aberrant signaling pathways could hold promise in alleviating subchondral bone alterations and mitigating OA severity.

### 4.2 sEV therapy on OA

sEV have emerged as promising tools in regenerative medicine and disease treatment. These nano-sized particles are released by various cell types and are enriched with bioactive molecules, including proteins, lipids, and nucleic acids. Their ability to deliver these molecules to target cells positions sEV as pivotal agents in intercellular communication, making them a focus of interest in therapeutic development. This review highlights the diverse therapeutic roles of sEV, focusing on their capacity to promote chondrogenesis, modulate inflammation, and stimulate angiogenesis and tissue repair.

#### 4.2.1 Promotion of chondrogenesis and cartilage repair and regeneration

They are critical challenges in orthopedic medicine due to the avascular and aneural nature of cartilage tissue. sEV have demonstrated a remarkable ability to support chondrogenesis, making them a promising therapeutic option for cartilage-related disorders such as OA.

Delivery of chondroprotective molecules in sEV derived from MSCs are particularly notable for their role in cartilage repair. They are enriched with chondroprotective factors, including SOX9 mRNA and specific microRNAs, such as miR-140, which are key regulators of chondrogenesis (LIN et al., 2021). SOX9, a transcription factor, is critical for cartilage formation as it regulates the expression of cartilage-specific extracellular matrix proteins, such as collagen type II and aggrecan. The presence of SOX9 mRNA in sEV ensures the transfer of this essential regulatory molecule to recipient cells, thereby enhancing cartilage repair mechanisms (Li et al., 2024c).

Similarly, miR-140 has been identified as a cartilage-specific microRNA that plays a pivotal role in maintaining cartilage homeostasis. Studies have shown that sEV enriched with miR-140 promote the proliferation and differentiation of chondrocytes while simultaneously inhibiting the expression of matrix-degrading enzymes such MMPs. This dual action ensures the preservation and repair of cartilage tissue, positioning sEV as a potent therapeutic tool (Ragni et al., 2022a).

Additionally, Scalzone et al. have proposed other factors such as BMP-2 and TGF-β3 found in sEV from BM-MSCs, that contribute to cartilage regeneration by enhancing chondrocyte differentiation and extracellular matrix deposition (Scalzone et al., 2024) (Table 1).

**TABLE 1:** Key Factors in sEV with Therapeutic Potential for OA treatment.

Therapeutic role	Factor	Function	References
Promotion of Chondrogenesis Cartilage repair and regeneration	SOX9 mRNA	Regulates cartilage matrix protein expression	Li et al. (2024c)
	miR-140	Promotes chondrocyte proliferation and inhibits MMPs	LIN et al. (2021), Pfeifer et al. (2024)
	BMP-2	Enhances chondrocyte differentiation and ECM deposition	Cheng et al. (2024)
	TGF-β3	Regulates chondrogenesis and cartilage repair	Scalzone et al. (2024)
Modulation of Inflammation Chronic	TGF-β	Inhibits pro-inflammatory pathways	Ruiz et al. (2020)
	IL-10	Suppresses TNF-α and IL-1β, reducing inflammation	Zhang et al. (2021), Wu et al. (2024b)
	IL-4	Promotes anti-inflammatory macrophage polarization	Li et al. (2022)
	IL-13	Reduces pro-inflammatory cytokine expression	Nguyen et al. (2022), Mohd Noor et al. (2024)
Stimulation of Angiogenesis and Tissue Repair	VEGF	Stimulates endothelial cell proliferation and migration	Genestra et al. (2023)
	HGF	Enhances angiogenesis and supports vascular survival	Ragni et al. (2024)
	FGF-2	Promotes endothelial cell growth and new vessel formation	Forteza-Genestra et al. (2024)

SOX9 (Transcription factor SOX-9); BMP-2 (Bone Morphogenetic Protein 2); TGF-β3 (Transforming Growth Factor-β3); IL-10 (Interleukin-10); IL-4 (Interleukin-4); IL-13 (Interleukin-13); VEGF (Vascular Endothelial Growth Factor); HGF (Hepatocyte Growth Factor); FGF-2 (Fibroblast Growth Factor-2).

#### 4.2.2 Enhancement of extracellular matrix production

A critical mechanism through which sEV promote chondrogenesis is by enhancing the synthesis of ECM components. sEV transport a wide array of bioactive molecules, including growth factors, cytokines, and miRNAs, which modulate cellular behavior and stimulate the production of essential ECM constituents such as type II collagen and glycosaminoglycans (Gendron et al., 2003; Siebelt et al., 2014). Notably, miR-140, a cartilage-specific miRNA frequently enriched in sEV, has been shown to promote chondrocyte proliferation while inhibiting the expression of matrix-degrading enzymes such as MMPs (LIN et al., 2021; Pfeifer et al., 2024). Similarly, bone morphogenetic protein-2 (BMP-2), another cargo molecule identified in therapeutic sEV, plays a pivotal role in enhancing chondrocyte differentiation and ECM deposition (Cheng et al., 2024).

In addition, Otahal et al. demonstrated that this ability to enhance ECM synthesis underscores the therapeutic promise of plasma- and serum-derived sEV in treating cartilage-related disorders (Otahal et al., 2020; 2021).

For example, sEV derived from induced pluripotent stem cells (iPSCs) have been shown to significantly boost ECM production in both *in vitro* and *in vivo* cartilage repair models. Hsueh et al. demonstrated that iPSC-derived sEV significantly enhanced ECM production in both *in vitro* and *in vivo* OA models. These sEV promoted primary chondrocyte proliferation, reduced premature senescence, and restored collagen II synthesis, while suppressing matrix-degrading enzymes like MMP-13 and ADAMTS-5. In a rabbit anterior cruciate ligament transection (ACLT)-induced OA model, intraarticular injection of iPSC-sEV ameliorated cartilage damage and preserved tissue integrity (Hsueh et al., 2023).

Further advancements have been achieved using MSCs differentiated from iPSCs as a source of sEV. Feng et al. developed engineered sEV derived from iPSC-MSCs, modified with a cartilage-targeting peptide and loaded with siRNA against MDM2. These sEV selectively targeted and eliminated senescent chondrocytes via modulation of the MDM2-p53 pathway, restoring ECM metabolic homeostasis and enhancing collagen II expression. The therapeutic effect was confirmed in both post-traumatic and age-related OA mouse models, as well as in *ex vivo* cultured human OA cartilage explants (Feng et al., 2025).

To overcome the challenge of limited bioavailability following intraarticular delivery, Yang et al. employed a novel injectable Diels–Alder crosslinked hyaluronic acid/PEG (DAHP) hydrogel to deliver MSC-sEV derived from iPSCs. This hydrogel ensured sustained release and preserved the functional integrity of sEV, resulting in enhanced cartilage repair and ECM regeneration *in vivo*. The study provided a clinically relevant platform that prolongs joint retention of therapeutic vesicles and supports their gradual release within the synovial environment (Yang et al., 2021).

Collectively, these studies emphasize the capacity of iPSC-derived MSC-sEV to not only stimulate ECM synthesis but also modulate chondrocyte senescence and matrix catabolism. Through innovative delivery systems and bioengineering, sEV emerge as potent, cell-free therapeutic agents with high translational potential in osteoarthritis treatment.

#### 4.2.3 Modulation of inflammation chronic

It is a hallmark of many degenerative diseases, including osteoarthritis and rheumatoid arthritis. The ability of sEV to modulate inflammatory responses makes them an attractive therapeutic candidate for managing these conditions.

sEV carry a diverse array of anti-inflammatory molecules, including cytokines and growth factors such as TGF-β and IL-10. TGF-β is a multifunctional cytokine that plays a critical role in immune regulation and tissue repair. By inhibiting the activation of pro-inflammatory pathways, TGF-β in sEV helps reduce synovial inflammation, a major driver of cartilage degradation in osteoarthritis (Ossendorff et al., 2023).

Similarly, IL-10 is a potent anti-inflammatory cytokine that suppresses the production of pro-inflammatory mediators such as TNF-α and IL-1β. The presence of IL-10 in sEV ensures the inhibition of these harmful inflammatory pathways, creating a more conducive environment for tissue repair and regeneration. In addition, factors such as IL-4 and IL-13 found in sEV contribute to immune modulation by promoting an anti-inflammatory macrophage phenotype and reducing pro-inflammatory cytokine expression (Nakamachi et al., 2023; Mohd Noor et al., 2024) (Table 1).

#### 4.2.4 Stimulation of angiogenesis and tissue repair

The repair and regeneration of damaged tissues frequently requires the re-establishment of vascular networks to ensure a sufficient supply of oxygen, nutrients, and cellular components necessary for repair. In this context, sEV have been shown to play a critical role in stimulating angiogenesis and facilitating tissue repair, making them a valuable therapeutic tool in regenerative medicine.

Angiogenic Factors in sEV are enriched with pro-angiogenic factors such as vascular endothelial growth factor (VEGF) and hepatocyte growth factor (HGF). VEGF is a key regulator of angiogenesis, promoting the proliferation and migration of endothelial cells to form new blood vessels. The presence of VEGF in sEV ensures the stimulation of angiogenic processes, which are critical for tissue repair and regeneration (Shang et al., 2021) (Table 1).

HGF, another important factor found in sEV, plays a dual role in promoting angiogenesis and enhancing tissue repair. HGF stimulates the migration and proliferation of endothelial cells while also promoting the survival of existing vascular structures. Additionally, fibroblast growth factor-2 (FGF-2), found in sEV, further enhances endothelial cell proliferation, and supports the formation of new blood vessels (Thomas et al., 2022) (Table 1).

Although articular cartilage is inherently avascular, a feature that is essential for its unique biomechanical and immune-privileged properties, the role of angiogenesis becomes increasingly relevant in the pathogenesis of OA. During OA progression, aberrant neovascularization has been observed in previously avascular regions such as the deep layers of articular cartilage and the osteochondral junction (Ragni et al., 2022b; Genestra et al., 2023; Forteza-Genestra et al., 2024).

Moreover, vascular remodeling in the subchondral bone and synovial membrane plays a critical role in OA. In the subchondral bone, increased angiogenesis is associated with bone marrow lesions, sclerosis, and osteophyte formation (Ragni et al., 2022b; Forteza-Genestra et al., 2024). Similarly, neovascularization within the synovium can sustain local inflammation and further disrupt joint homeostasis.

Given this context, the modulation of angiogenesis by sEV offers a promising therapeutic avenue in OA. Rather than indiscriminately promoting neovascularization, targeted delivery of sEV could support vascular remodeling in beneficial zones such as the subchondral bone while avoiding detrimental vascular invasion into cartilage.

#### 4.2.5 Enhancement of subchondral bone remodeling

In addition to promoting angiogenesis, sEV also play a role in subchondral bone remodeling. Subchondral bone, located beneath the cartilage, plays a critical role in supporting cartilage health and function. Damage to this structure is a common feature of OA and other joint disorders. sEV derived from MSCs have been shown to promote the repair and remodeling of subchondral bone by delivering bioactive molecules that stimulate osteoblast activity and inhibit osteoclast-mediated bone resorption. This ability to restore subchondral bone integrity further underscores the therapeutic potential of sEV in joint repair (Niedermair et al., 2020).

These findings collectively highlight the significant potential of sEV in regenerative medicine, particularly for cartilage repair, immune modulation, and tissue regeneration.

### 4.3 OA pre-clinical sEV-based studies

Recent pre-clinical studies have explored the therapeutic potential of sEV derived from various cell sources in OA models. These studies provide compelling evidence for the efficacy of sEV in mitigating OA progression, reducing inflammation, and promoting cartilage regeneration (Table 2).

**TABLE 2:** The most relevant pre-clinical studies about therapeutic potential of sEV on OA.

sEV source	Year	Pre-clinical model	Findings	References
AD-MSCs	2020	Monosodium iodoacetate (MIA)- induced and DMM were conducted in male rat (seven-week-old)	Intra-articular injection of sEV attenuated OA progression and protected cartilage from degeneration	Woo et al. (2020)
	2022	ACLT surgery was conducted on male C57 mice	sEV from curcumin primed AD-MSCs inhibit chondrocyte apoptosis and OA progression	Xu et al. (2022)
	2022	DMM surgery was conducted on male C57BL/6 mice (10-week-old)	IGF-1-ADSCs delay the progression of OA by reduction of cartilage destruction and the increase of anabolic markers of chondrocytes	Wu et al. (2022)
	2024	TMJ OA group, WT and Ggpps cKO mice (10-weeks old) were treated with unilateral anterior crossbite models (UAC)	Obesity-induced TMJ OA progression is exacerbated by AD-MSC-sEV from obese mice, with miR-3074-5p playing a key role, and *Ggpps* gene knockout in adipose tissue inhibiting this effect	Li et al. (2024a)
	2023	CIOA model was conducted on male C57BL/6 mice (8-week old)	DM4-sEV significantly suppressed the osteochondral degeneration	Hanai et al. (2023)
	2024	CIOA was performed on murine model (10-week-old)	Senescent sEV do not exert the chondroprotective effect	Jérémy et al. (2024)
SF-treated AD-MSCs	2021	SF from OA (Kellgren and Lawrence III–IV grade) patients (between 52 and 87 years) (women and men)	sEV from AD-MSCs change the miRNA composition and their therapeutic properties as: 1) immune potential properties (M2 macrophage polarization, T cell proliferation inhibition and T reg expansion enhancement); 2) cartilage protection	Ragni et al. (2021b)
AD- and BM-MSCs	2021	Ciprofloxacin treatment by gastric gavage in female BALB/c mice (three-week-old)	sEV from BM-MSCs shown more cartilage repair capacity than sEV from AD-MSCs	Fazaeli et al. (2021)
BM-MSCs	2020	IL-1β treatment in cartilage explants from C57BL/6 mice (two-week-old)	TFGBI mRNA and protein contained in sEV shown a chondroprotective factor and anabolic regulator of cartilage homeostasis	Ruiz et al. (2020)
	2021	Temporomandibular joint osteoarthritis (TMJOA) using chemical method modelling-collagenase injection on New Zealand female and male rabbits (12- to 18-week-old)	sEV from BM-MSCs promote cartilage reconstruction in TMJOA through autotaxin-YAP signalling axis	Wang et al. (2021b)
	2021	ACLT was conducted on female Sprague-Dawley rats (7–8 weeks old)	Hypoxia pre-treatment on sEV protect cartilage from degeneration and slow down the progression of OA. They can promote the chondrocyte proliferation, migration, and apoptosis inhibition through the miR-216a-5p/JAK2/STAT3 signalling pathway	Rong et al. (2021)
	2021	Collagenase II was injected into the knee joint cavity of male Sprague-Dawley rats (10-weeks old)	lncRNA malat-1 contained in sEV promote chondrocyte proliferation, alleviate chondrocyte inflammation and cartilage degeneration	Pan et al. (2021)
	2021	Collagenase II was injected into the knee joint cavity of C57BL/6 mice (9-weeks old)	sEV-circHIPK3 modulate the chondrocyte proliferation, migration induction and the apoptosis inhibition through miR-124-3p/MYH9 axis	Li et al. (2021)
	2022	DMM surgery was conducted on adult male C57BL/6 mice (5–6 months old)	NEAT1 delivery by BM-MSC-sEV activate the Sen2/Nrf2 axis via binding to miR-122-5p for protection against OA	Zhang and Jin (2022)
	2022	DMM surgery was conducted on adult male rats	sEV from TGFβ3-preconditioned BM-MSCs are enriched in miR-455 that promotes OA alleviation and cartilage regeneration by activation the SOX11/FOXO signaling pathway	Sun et al. (2022)
	2022	Modified Hulth method was conducted on female Sprague-Dawley rats (4-weeks old)	Hypoxia-preconditioned sEV promote cartilage repair by stimulating chondrocyte proliferation, migrarion and suppressing chondrocyte apoptosis through the miRNA-18-3p/JAK/STAT or miR-181c-5p/MAPK signaling pathway	Zhang et al. (2022)
	2022	DMM surgery conducted on C57BL/6 J male mice (8-weeks old)	MSC-derived EV-loaded miR-3960 downregulated PHLDA2 to inhibit chondrocyte injury via SDC1/Wnt/β-catenin	Ye et al. (2022)
	2023	ACLT was conducted on Sprague-Dawley male rats (8-weeks old)	BMSC-sEV reduced chondrocyte ferroptosis and prevented OA progression via disruption of the METTL3-m6A-ACSL4 axis	Cheng et al. (2024)
	2023	DMM was conducted on male C57BL/6J mice (10–12 weeks old)	MSC-sEV treatment in DMM-operated mice prevented pain-related behaviors by inhibiting sensory neuron hyperexcitability, without reducing joint damage	Ai et al. (2023)
	2023	ACLT was conducted on Wistar-Han male rats (12-weeks old)	MSC treatment negatively impacts metabolic mild OA, while MSC-EVs show potential but require optimization for effective therapy	Warmink et al. (2023)
	2024	ACLT was conducted on male C57BL/6 mice (68-weeks old)	sEV combined with ultrasmall superparamagnetic iron oxide particles, activate anabolic pathway, inhibit catabolic activities, and promote M2 polarization through the microRNA-99b/MFG-E8/NF-κB signaling axis	Wang et al. (2024)
	2024	DMM was conducted on male Sprague-Dawley rats (approximately 12 weeks old)	GMOCS-sEV hydrogel modulate TGFB1/Nrf2 pathway	Zhou et al. (2024)
	2024	DMM was conducted on male C57BL/6 mice (7-week-old)	Donor age is a critical determinant in the therapeutic potential of BM-MSC-derived sEV	Wakale et al. (2024)
	2024	ACLT was conducted on male BALB/c mice (12-week-old)	TGF-β inhibitor-loaded sEV block the activation of abnormal ossfication	Jing et al. (2024)
	2024	Knee injury by surgery was conducted on male Sprague-Dawley rats (10-week-old)	sEV can be incorporated into polysaccharides SA-HA composite hydrogel and they promote MSC or chondrocyte proliferation, stimulation of MSCs, chondrocyte migration, and inhibition of chondrocyte hypertrophy and concomitant promotion of cartilage matric component expression and suppression of cartilage matrix degradation	Li et al. (2024b)
	2024	ACLT surgery was conducted on Sprague-Dawley rats	The strategy the microneedle-delivery polydopamine-sEV could prevent cartilage degradation by PI3K/Akt/mTOR pathway	Li et al. (2024d)
	2024	Mechanical load was conducted on male C57BL/6 mice (5-8-week-old)	miR-125a-5p contained in sEV suppress chondrocyte degeneration via targeting E2F2	Xia et al. (2021)
	2024	MIA induction was conducted on Sprague-Dawley rats (8-week-old)	Puerorin-loaded in sEV increase the solubility of Puerorin and stimulate the proliferation, migration of chondrocyte and cartilage repair	Zhang et al. (2024b)
Co-aggregation of BM-MSC/chondrocyte	2022	MIA-induced OA was performed on adult male Wistar rats	sEV from co-aggregation of BM-MSC/Chondrocyte have a high therapeutic potential to OA	Esmaeili et al. (2022)
Chondrocytes	2020	Destabilization of the meniscus (DMM) in female C57B/L10 mice (eight-week-old)	Intra-articular injections of sEV stimulated chondrocyte proliferation and migration, leading to enhanced cartilage repair and attenuation of OA severity	Wang et al. (2020)
	2024	Articular cartilage defect was conducted on adult male Sprague Dawley rats	Chondrocyte-sEV carrying lncRNA LOC102546541 act as a ceRNA for MMP13, downregulating miR-632 and enhancing MMP13-mediated extracellular matrix degradation	Lai et al. (2024)
	2024	ACLT was conducted on Sprague-Dawley male mice (8-week-old)	sEV from chondrocytes promotes matrix anabolism and inhibit inflammatory response partially via blockinh STAT3 activation	Feng et al. (2024a)
	2024	ACLT was conducted on C57BL/6 male rats (more than 2 months)	sEV from chondrocytes with MMP13 enrichment can attenuate inflammation, promoted chondrocyte proliferation, collagen synthesis, and reduce apoptosis	Yan et al. (2024)
	2024	MIA was conducted on male Sprague-Dawley Rats (8-week-old)	sEV from chondrocytes downregulate inflammatory cytokines (TNF, IL-17) and upregulated pathways for cellular proliferation, migration, and metabolism	Chen et al. (2024)
	2025	ACLT surgery was conducted on Sprague-Dawley (10-14-week-old)	sEV from IL-1β-treated CH augmentee hyaline cartilage, reducing synovium inflammation, and promoting trabecular formation in the subchondral bone during the early stage of pathology	Xu et al. (2025)
ESCs	2024	ACLT surgery was conducted on c C57BL/6 male mice (12-week-old)	ESC-sEV alleviate non-early-stage OA progression by rejuvenating senescent chondrocytes via FOXO1A-autophagy	Chen et al. (2024), Feng et al. (2024b)
Garlic	2025	ACLT and DMM surgery were conducted on C57BL/6 mice	sEV from garlic alleviate the sensitivity and ameliorate the joint destruction	Liu et al. (2024b)
PESCs	2021	Women and men patients with grade II/III OA (Kellgren Lawrence (KL) grading scale) (>18 years old)	This work shown the safety and improvement in their pain, function, quality of life with the intraarticular injection of cell-free PESCs in patients	Gupta et al. (2021)
SF	2021	Women and men patients with knee joint disorders (primary OA) (>18 years old)	The intraarticular injection of HA-sEV could be involved in the immunosuppressive capacity	Mustonen et al. (2021)
	2022	Carpal osteochondral fragment-exercise model on horse (7-years old)	SF-derived sEV after the integrin α10-MSC administration mediate the effect of cell therapy	Clarke et al. (2022)
iPSC-derived MSCs	2021	ACLT in combination with partial medial meniscectomy in male Sprage-Dawley rats (8-week-old)	sEV from iPSC-derived MSCs in a Diels–Alder crosslinked HA/PEG hydrogel could enhanced the effectivity of sEV	Yang et al. (2021)
iPSCs	2022	ACLT was conducted on female New Zealand white rabbits	iPSC-sEV protect chondrocytes by promoting cell proliferation, preventing senescence, maintaining collagen II and matrix enzyme balance, reducing cell death in inflammatory conditions, and alleviating cartilage damage	Hsueh et al. (2023)
	2025	ACLT surgery was conducted on male mice (8-week-old)	sEV with peptide inhibitor of p53 and siRNA of MDM2 eliminate senescence cells (senolytic), maintain matrix metabolic homeostasis	Feng et al. (2025)
S-MSCs	2021	Destabilization of the medial meniscus was conducted on male Sprague-Dawley rats	Kartogenin contained in sEV promote cartilage regeneration	Xu et al. (2021)
	2021	DMM surgery was conducted on male C57BL/6 mice (10-weeks old)	LPS-pre-treatment sEV improved cartilage damage through inhibition of ADAMTS-5 to avoid the degradation of aggrecan and COL2A1	Duan et al. (2021)
	2021	OA was induced by transecting the medial collateral ligament, removing the medial meniscus at its narrowest point, and shortening the anterior cruciate ligament in 12-week-old male Sprague-Dawley rats	cicRNA3503-sEV alleviate inflammation-induced apoptosis and the imbalance between ECM synthesis and degradation	Tao et al. (2021)
	2021	DMM surgery was conducted on adult male C57BL/6 mice	sEV-contained miR-26a-5p inhibit apoptosis, inflammation, and cartilage damage through PTEN inhibition	Lu et al. (2021)
	2020	DMM in male C57 mice (eight-week-old)	S-MSC-sEV-encapsulated miR-31 can modulate the balance between the synthesis and degradation of the cartilage extracellular matrix, leading to amelioration of OA symptoms	Wang et al. (2020)
	2024	CIOA was performed on male Sprague-Dawley rats (9-week-old)	Jintiange capsules improve the degeneration of joint cartilage and produce changes in mRNA and miRNA contained in sEV from S-MSCs	Zhongying et al. (2024)
DP-SCs	2021	Iodoacetic acid intraarticular injection on male Sprague-Dawley (8 weeks old)	miR-140-enriched sEV inhibit the apoptosis of chondrocytes and promoting cartilage repair	LIN et al. (2021)
PPD-MSCs	2021	ACLT was conducted on male C57BL mice (68-weeks old)	Reversing the surface charge of sEV by εPL-PEG-DSPE, show more intra-articular bioavailability and efficacy than native sEV	Feng et al. (2021)
UC-MSCs	2021	Anterior cruciate ligament transection (ACLT) in male Sprague-Dawley rats (8-week-old)	sEV from UC-MSCs stimulates chondrocyte activity and matrix remodelling processes in an inflammatory environment	Tang et al. (2021)
	2022	DMM surgery was conducted on male rats (between 6 and 8 weeks old)	sEV can alleviate cartilage degradation during the OA progression through PI3K/Akt signalling pathway by miRNAs to promote polarization of M2 macrophages	Li et al. (2022)
	2022	DMM surgery conducted on male C57BL/6 mice (8-week-old)	UC-MSCs-derived EVs reduced NLRP3 mRNA m6A methylation through miR-1208 and METTL3, inhibiting pro-inflammatory factors and cartilage ECM degradation, thus alleviating OA progression in mice	Zhou et al. (2022)
	2022	Intra-articular injection of human chondrocytes in knee was conducted on male Wistar rats (12-months old)	MSC-miR-21−-derived sEV show a higher therapeutic potential in comparison with MSCs-miR-21-. They ameliorate the systemic inflammation through inactivation of ERK1/2 pathway	Morente-López et al. (2022)
	2022	ACLT was conducted on Sprague-Dawley male rats (8-weeks old)	Let-7e-5p, present in UC-MSC-sEV, has been identified as a potential key molecule for promoting cartilage regeneration by regulating STAT3 and IGF1R levels	Chen et al. (2022a)
	2023	MIA injected intraperitoneally was conducted on Sprague-Dawley male rats (8-weeks old)	UC-EVs maintain chondrocyte homeostasis and protect articular cartilage from damage in OA rat via miR-223/NLRP3/pyroptosis axis	Liu et al. (2023b)
	2024	Meniscectomy model in male Wistar rats (between 12 and 14-week-old)	sEV shown a great anti-inflammatory effect through downregulation inflammatory markers (TNF-α, iNOS) and increase Arg-1 expression in macrophages and synovial fibroblasts	Klyucherev et al. (2024)
	2025	CIOA was conducted on male and female C57BL/6J mice (8–12-week-old) and clinical study with human	It is the first clinical study of sEV intra-articular injection, it shows regenerative and anti-inflammatory	Figueroa-Valdés et al. (2025)
Platelet and UC-MSCs	2023	Healthy human cartilage explants from talus and calcaneus bones cultured in OA-like condition	sEV from platelet are a promising treatment in cartilage regeneration	Genestra et al. (2023)
Platelets and UC-MSCs	2024	MIA was conducted on male and female Wistar rats (8-week-old)	sEV from platelets demonstrate more OA reversion in knee joints than sEV from UC-MSCs	Forteza-Genestra et al. (2024)
Bovine milk	2022	Cartilage explants from anonymous OA donors undergoing total knee replacement	sEV from bovine milk contain TGF-β and miR-148a, two essential regulators for maintaining chondrocyte homeostasis and protection against cartilage destruction	Pieters et al. (2022)
	2023	DMM was conducted on male C57BL/6J mice (7 weeks old	bovine milk-derived sEV can alleviate OA progression by restoring matrix homeostasis and reshaping the gut microbiota	Liu et al. (2023a)
Serum	2022	ACLT surgery was conducted on female Wistar rats (8-week-old)	sEV from serum can regulate the Trim14/NF-κB/IFN-β axis of the innate immune response by delivering miR-150-3p to chondrocytes to maintain joint homeostasis, repair articular cartilage injury, and delay OA progression	Wang et al. (2022)
	2024	MIA injection was conducted on adult male Sprague-Dawley rats	miR-133a-3p-containing sEV from serum modulate the downstream SIRT1/NF-κB pathway-mediated pyroptotic cell death and inflammation	Tang et al. (2024b)
Synoviocytes	2022	DMM surgery was conducted on male Sprague-Dawley rats (4-weeks old)	EVs produced by synoviocyte from OA rats contained miR-382-5p, which might reduce cell viability and proliferation, and promote cell apoptosis by targeting PTEN	Lu et al. (2022)
	2023	DMM was conducted on male C57BL/6 mice (10-weeks old)	sEV delivery of miR-25-3p reduced chondrocyte pyroptosis by inhibiting CPEB1, with CPEB1 overexpression reversing this effect in knee OA.	Wang and Sun (2023)
	2023	ACLT and resection of medial menisci (MMx) were conducted on male Sprague Dawley rats	miR-214-3p-sEV from synoviocites can ameliorate chondrocyte inflammation and degeneration of cartilage tissues	Lai et al. (2023)
	2024	DMM was conducted on mice (8 weeks old)	sEV from synoviocytes containing hydrogel microspheres could deliver SOD3 to cartilage and significantly mitigate OA progressio	Cao et al. (2024a)
IFP-MSCs	2023	MIA-induced OA was conducted on male Sprague Dawley rats (10-week old)	CD10-High sEV exhibit immunomodulatory miRNA properties with potent chondroprotective and anabolic effects	Kouroupis et al. (2023)
	2024	DMM surgery was conducted on C57 mice male (9-week-old)	TNF-α preconditioning constitutes an effective and promising method for optimizing the therapeutic effects of IPFP-MSCs derived sEV through PI3K/AKT signaling pathway	Wu et al. (2024a)
	2024	DMM surgery was conducted on male C57BL/6 (12-week-old)	let-7b-5p and let-7c-5p contained in sEV increase Lamin B receptor expression, suppress chondrocyte senescence, and ameliorated the progression of experimental OA model	Cao et al. (2024b)
	2024	ACLT surgery was conducted on male C57BL/6 mice (10-week-old)	IPFP-MSC-derived sEV could delay OA progression by alleviating pain and suppressing cartilage degeneration, osteophyte formation, and synovial inflammation	Liu et al. (2024a)
Macrophages	2023	Collagenase-induced OA (CIOA) and ACLT were conducted on male C57BL/6 mice (12-weeks old)	Inhibiting caspase 11-mediated pyroptosis reduced inflammatory macrophage-derived EV effects on chondrocytes and alleviated OA in mice, offering a potential therapeutic target	Ebata et al. (2023)
	2024	ACLT was conducted on female and male Sprague-Dawley rats (Thirty 2-month-old)	sEV from macrophages loaded in an injectable thermosensitive hydrogel for sustained sEV release, improve lymphatic drainage	Song et al. (2024)
	2024	DMM was conducted on male C57BL/6J (10-week-old)	sEV from macrophages alleviate mitochondrial dysfunction induced chondrocyte damage via ENPP1-induced suppression of the AKT/Notch2 pathway	Tang et al. (2024a)
	2024	DMM surgery and CIOA were conducted on male C57BL/6J mice (eight-week-old)	sEV from M2-macrophages decrease macrophage accumulation, repolarized macrophages from M1 to M2 phenotype, mitigated synovitis, reduced cartilage degradation, alleviate subchondral bone damage	Yuan et al. (2024)
Macrophages and IPFP-MSCs	2024	Sprague–Dawley rats (30 six-week-old)	This study provides and injectable, integrated delivery system with spatiotemporal control release of dual sEV	Li et al. (2024c)
Neutrophils	2024	CIOA was induced by two intra-articular injections, was conducted on male C57BL/6 mice (8-week-old)	sEV from neutrophils have high levels of SFRP5, that reduce catabolic process	Kitahara et al. (2024)
TMSCs	2024	DMM surgery was conducted on Sprague–Dawley rats (8-week-old)	The TGF-β1-overexpressed sEV contributed to maintaining homeostasis in the cartilage microenvironment and protecting the cartilage	Kim et al. (2024)
OUMS-27	2024	C57BL/6J mice	sEV can take up by cells within the cartilage, into joint fluid	Ohtsuki et al. (2024)

Studies utilizing MSC-sEV have demonstrated promising chondroprotective effects. For instance, BM-MSCs sEV have been shown to modulate key signaling pathways such as JAK2/STAT3, SOX11/FOXO, and PI3K/Akt to promote chondrocyte proliferation, migration, and survival, as well as inhibit apoptosis and inflammation (Fazaeli et al., 2021; Hotham et al., 2021; Pan et al., 2021; Ragni et al., 2022b; Li Z. et al., 2024; Scalzone et al., 2024; Tang X. et al., 2024). Notably, pre-conditioning strategies such as hypoxia treatment or cytokine stimulation have enhanced the therapeutic efficacy of these sEV, leading to improved cartilage repair and reduced OA severity (Zhang et al., 2022). Additionally, sEV from BM-MSCs have been found to alleviate oxidative stress, modulate immune cell activity, and regulate the extracellular matrix composition, further contributing to cartilage protection and repair (Hotham et al., 2021; Wang X. et al., 2021; Contentin et al., 2022).

Similarly, AD-MSCs sEV have exhibited significant anti-inflammatory and cartilage-protective properties. Intra-articular injections of sEV from AD-MSCs have resulted in reduced cartilage destruction, increased expression of anabolic markers, and inhibition of inflammatory pathways (Ragni et al., 2020; Cavallo et al., 2022; Hanai et al., 2023). Some studies have further optimized sEV function through priming with bioactive molecules such as curcumin and insulin-like growth factor-1 (IGF-1), leading to enhanced therapeutic effects (Wu et al., 2022). Additionally, AD-MSC-derived sEV have been observed to modulate macrophage polarization, enhancing the regenerative environment within the joint (Ragni et al., 2021b).

In addition to MSC-derived sEV, synoviocyte-derived sEV have shown potential in OA therapy by delivering specific microRNAs that modulate inflammatory and apoptotic pathways (Filali et al., 2022; Wang and Sun, 2023; Ohtsuki et al., 2024). For example, miR-214-3p-enriched sEV from synoviocytes have demonstrated efficacy in reducing chondrocyte inflammation and preventing cartilage degradation (Ragni et al., 2019c). Other studies have indicated that synoviocyte-derived sEV may regulate immune responses by influencing T-cell activity and cytokine release, further contributing to OA mitigation (Farina et al., 2022; Zeng et al., 2022; Kouroupis et al., 2023; Nakamachi et al., 2023; Ossendorff et al., 2023; Mohd Noor et al., 2024).

sEV derived from UC-MSC are the most extensively studied sEV-based therapy for OA, showing greater potential compared to sEV from other sources such as bone marrow or adipose tissue (Tang et al., 2021; Morente-López et al., 2022). A recent pioneering clinical study has demonstrated that UC-MSC-derived sEV are not only safer and more secure than MSCs themselves but also more effective in modulating inflammation (Tang et al., 2021; Li et al., 2022; Nguyen et al., 2022; Zhou et al., 2022; Klyucherev et al., 2024; Figueroa-Valdés et al., 2025). Their therapeutic effect is largely mediated through the ERK signaling pathway, which regulates immune responses and cartilage regeneration (Morente-López et al., 2022).

Chondrocyte-derived sEV have also been extensively studied for their regenerative potential. These sEV have been reported to promote extracellular matrix synthesis and inhibit matrix degradation through mechanisms such as MMP13 modulation and STAT3 pathway inhibition (Yan et al., 2024). Furthermore, their role in maintaining chondrocyte phenotype and preventing hypertrophic differentiation highlights their potential in OA treatment. Research has suggested that chondrocyte-derived sEV may also interact with other joint-resident cells, such as fibroblasts and synoviocytes, to create a more favorable microenvironment for cartilage repair.

Other emerging sources of sEV, including those from iPSCs, macrophages, and neutrophils, have also exhibited promising OA-therapeutic properties. iPSC-derived MSCs sEV, for instance, have been shown to enhance cartilage repair and delay OA progression by maintaining chondrocyte homeostasis and reducing senescence (Hsueh et al., 2023). Moreover, macrophage-derived sEV have been implicated in immunomodulatory functions, promoting M2 macrophage polarization and mitigating synovitis (Song et al., 2024; Wang et al., 2024). Neutrophil-derived sEV, particularly those enriched with anti-inflammatory molecules such as secreted frizzled-related protein 5 (SFRP5) (Kitahara et al., 2024), have demonstrated the ability to counteract catabolic processes in the joint, further supporting their potential use in OA treatment.

There are some alternative sources of sEV offer a complementary approach to cell-derived extracellular vesicle therapies, expanding the possibilities for OA treatment. Bovine milk and garlic-derived sEV provide additional benefits by promoting cartilage homeostasis, regulating the gut microbiota, and reducing pain sensitivity (Pieters et al., 2022; Liu Q. et al., 2023; Liu et al., 2024 Y.).

The delivery of sEV has also been optimized through innovative approaches such as hydrogel encapsulation, microneedle delivery systems, and surface charge modifications, which have improved intra-articular retention and bioavailability (Tao et al., 2021; Yang et al., 2021; Li et al., 2024b; Li et al., 2024 Z.; Song et al., 2024; Zhou et al., 2024). Hydrogel systems, in particular, have been engineered to provide sustained release of sEV, extending their therapeutic effects within the joint. Additionally, researchers have explored the co-administration of sEV with growth factors or nanoparticles to enhance their regenerative properties. These advancements suggest that sEV-based therapies hold great promise for OA treatment by harnessing their regenerative, anti-inflammatory, and chondroprotective capabilities.

Moreover, recent pre-clinical studies have highlighted the importance of donor characteristics and pre-conditioning strategies in determining the efficacy of sEV (Visconte et al., 2024). For example, donor age has been identified as a critical factor influencing the therapeutic potential of BM-MSC-derived sEV, with younger donors exhibiting greater chondroprotective effects. Additionally, inflammatory pre-conditioning or genetic modifications of sEV-producing cells have been explored to enhance their bioactivity, offering new avenues for optimizing sEV-based therapies (Wakale et al., 2024).

Overall, pre-clinical studies indicate that sEV could serve as a viable therapeutic option for OA, paving the way for future clinical trials to further validate their safety and efficacy in human subjects. Given their ability to modulate key signaling pathways, regulate immune responses, and promote cartilage repair, sEV-based interventions represent a promising frontier in regenerative medicine for OA treatment.

### 4.4 sEV cargo as potential biomarkers in OA

The identification of reliable biomarkers for OA diagnosis, prognosis, and therapeutic monitoring remains a critical challenge. sEV have emerged as promising candidates due to their ability to encapsulate and transport bioactive molecules, including microRNAs, proteins, lipids, and cytokines, which reflect the pathological state of OA.

#### 4.4.1 Nucleic acid cargo

Several studies highlight the role of miRNA profiles within sEV in OA. For instance, miRNA profiling of sEV derived from synovial fluid, cartilage, and BM-MSCs has revealed disease-specific signatures (Ragni et al., 2019b; 2019a; 2021a; Sanjurjo-Rodríguez et al., 2021; Filali et al., 2022; Ning et al., 2023; Asghar et al., 2024; Visconte et al., 2024). Ragni et al. have proposed the most abundant sEV-miRNAs from several OA affected-tissues as potential protective and destructive biomarkers. In cartilage, miR-24-3p and miR-193b-3p as protective and miR-21-5p and miR-16-5p as destructive; in synovium, miR-29a-3p (protective) and miR-34a-5p (destructive) (Ragni et al., 2022b). Sanjurjo-Rodríguez et al. reported that sEV-BM-MSCs derived from OA patients are enriched on miRNA involved in chondrogenesis, including as miR-142-3p, miR-630, miR-29a-3p, and miR-145-5p (Sanjurjo-Rodríguez et al., 2021). *In vitro* approximation using OA synovial fibroblasts have shown that these cells produce sEV with high content in miRNA-associated with osteoclastogenic process as miR-182, miR-4472-2, miR-1302-3, miR-6720, miR-6087 and miR-4532 (Asghar et al., 2024). The limitation of the miRNA cargo in sEV as a potential biomarker is the inexistence of reference genes in pathological tissues and cells. To solve this weakness, Ragni et al. have reported that miR-103a-3p and miR-22-5p have been identified as stable reference genes in sEV from various joint tissues (Ragni et al., 2021a), suggesting their potential as normalization controls in OA biomarker studies (Ragni et al., 2019b; 2019a). Additionally, integrative analyses of cartilage-derived sEV and single-cell RNA sequencing have uncovered specific miRNAs associated with OA progression, further supporting their diagnostic relevance. Ning et al. have identified miR-7-5p as one of the cartilage-derived sEV, that targets genes involved in the activation of the ferroptosis process and cartilage degradation in OA (Ning et al., 2023). This miRNA could be used as biomarker and potential therapeutic target to avoid the pathology progression.

Expanding on these insights, Liebmann et al. demonstrated that engineering IFP-derived MSCs with an adeno-associated virus (AAV) vector encoding a CGRP antagonist (CGRP8–37) yields sEV (aCGRP IFP-MSC sEV) with potent anti-inflammatory and analgesic properties. These vesicles carried a distinct miRNA and protein cargo involved in immune regulation, pain modulation, and cartilage repair, including miR-21-5p, miR-23a-3p, and proteins like TIMP-2 and IL-10. Functionally, they promoted M2 macrophage polarization and reduced neuroinflammation (Liebmann et al., 2024).

Beyond miRNAs, Circular RNAs (circRNAs), a novel class of endogenous covalently linked RNA molecules, were characterized by a closed continuous loop. CircRNAs are abundant, widespread, and tissue specific. CircRNAs have primary played a role as microRNA sponges. As evolutionarily conserved small noncoding RNAs (ncRNAs), miRNAs could inhibit gene expression by binding the base with the 3′untranslated region (3′-UTR) of mRNAs. Lin et al. have proposed that circPARD3B was down enriched in sEV from OA synovial (Lin et al., 2022).

#### 4.4.2 Protein and lipid cargo

Proteomic analyses of sEV have revealed distinct protein cargo associated with OA severity and progression. iTRAQ proteomic analysis of exosomes derived from synovial fluid has identified disease-specific protein patterns (C3, C4B, APOM, MMP3, DPYSL2) that may serve as potential biomarkers (Wu X. et al., 2024). Similarly, OA modifies fatty acid signatures in synovial fluid from equine OA model and its sEV, indicating a metabolic shift that could be leveraged for early disease detection (Mustonen et al., 2023).

Inflammation plays a central role in OA pathogenesis, and sEV have been shown to carry key inflammatory mediators. Plasma-derived sEV carrying TNF-α have been identified as predictive biomarkers for knee OA progression (Zhang et al., 2021). Furthermore, immune-related plasma pathogenic EV subpopulations have been linked to OA progression, underscoring their potential as non-invasive biomarkers for disease monitoring (Zhang X. et al., 2024).

Surface marker profiling of plasma-derived sEV has provided valuable insights into OA severity. For instance, the expression levels of CD45, CD326, and CD56 on sEV correlate with OA stage, offering a novel diagnostic tool (Matejova et al., 2023). Additionally, tetraspanin profiles of serum sEV have been found to reflect functional limitations and pain perception in knee OA, demonstrating their utility in patient stratification and symptom monitoring (Mustonen et al., 2024).

The emerging evidence underscores the significant potential of sEV as biomarkers in OA. Their molecular cargo—ranging from miRNAs and proteins to lipids and inflammatory mediators—provides a comprehensive reflection of disease pathophysiology (Kolhe et al., 2020; Lin et al., 2022; Mustonen et al., 2022; Zhang et al., 2023). With advancements in high-throughput omics technologies and bioinformatics approaches, sEV-based biomarkers hold promise for improving OA diagnosis, prognostic assessment, and therapeutic response monitoring. Further validation in large-scale clinical studies will be essential to translate these findings into clinical practice.

### 4.5 Potential of sEV therapy in meniscus, tendons, ligaments, and muscle involvement in OA

Although the current literature on sEV therapy in OA predominantly focuses on cartilage, synovium, and subchondral bone, other periarticular structures such as the meniscus, ligaments, tendons, and muscles also play critical roles in joint homeostasis and OA progression.

The meniscus is essential for load distribution and joint stability. Meniscal damage is a well-established risk factor for OA, and the destabilization of the medial meniscus (DMM) model is widely used in preclinical OA research (Wang et al., 2020; Duan et al., 2021; Tao et al., 2021). While sEV have been tested in DMM-induced OA models, their direct regenerative or protective effects on meniscal tissue remain underexplored. Given the meniscus’s fibrocartilaginous nature, future studies should investigate whether sEVs can modulate meniscal cell metabolism, ECM remodeling, or inflammation.

Similarly, ligamentous structures, particularly the anterior cruciate ligament (ACL), are frequently involved in post-traumatic OA. Several studies in the review utilized the ACLT model (Rong et al., 2021; Cheng et al., 2024), which mimics joint instability and subsequent OA development. However, the potential of sEVs to enhance ligament healing or modulate ligament-derived inflammation has not been directly addressed. Given the regenerative potential of MSC-derived sEVs, their application in ligament repair warrants further investigation.

Tendons, though less frequently studied in OA models, are also susceptible to degeneration and inflammation in the OA joint environment. sEVs have shown promise in tendon healing in other musculoskeletal contexts, suggesting a translational opportunity for OA-related tendon pathologies.

Lastly, muscle weakness and atrophy, particularly of the quadriceps, are common in OA and contribute to joint dysfunction and pain. Although not a primary focus of OA sEV studies, the immunomodulatory and regenerative cargo of sEV, including miRNAs and growth factors, may have potential to support muscle regeneration and neuromuscular function, especially in advanced OA or post-surgical rehabilitation.

Incorporating these tissues into the sEV therapeutic landscape could provide a more holistic and functional approach to OA management, particularly in post-traumatic or multifactorial OA. Future studies should aim to characterize the effects of sEVs on these tissues and explore their potential in multitissue-targeted therapies.

## 5 Challenges and future perspectives

### 5.1 Challenges

#### 5.1.1 Isolation and characterization

**FIGURE 5:**
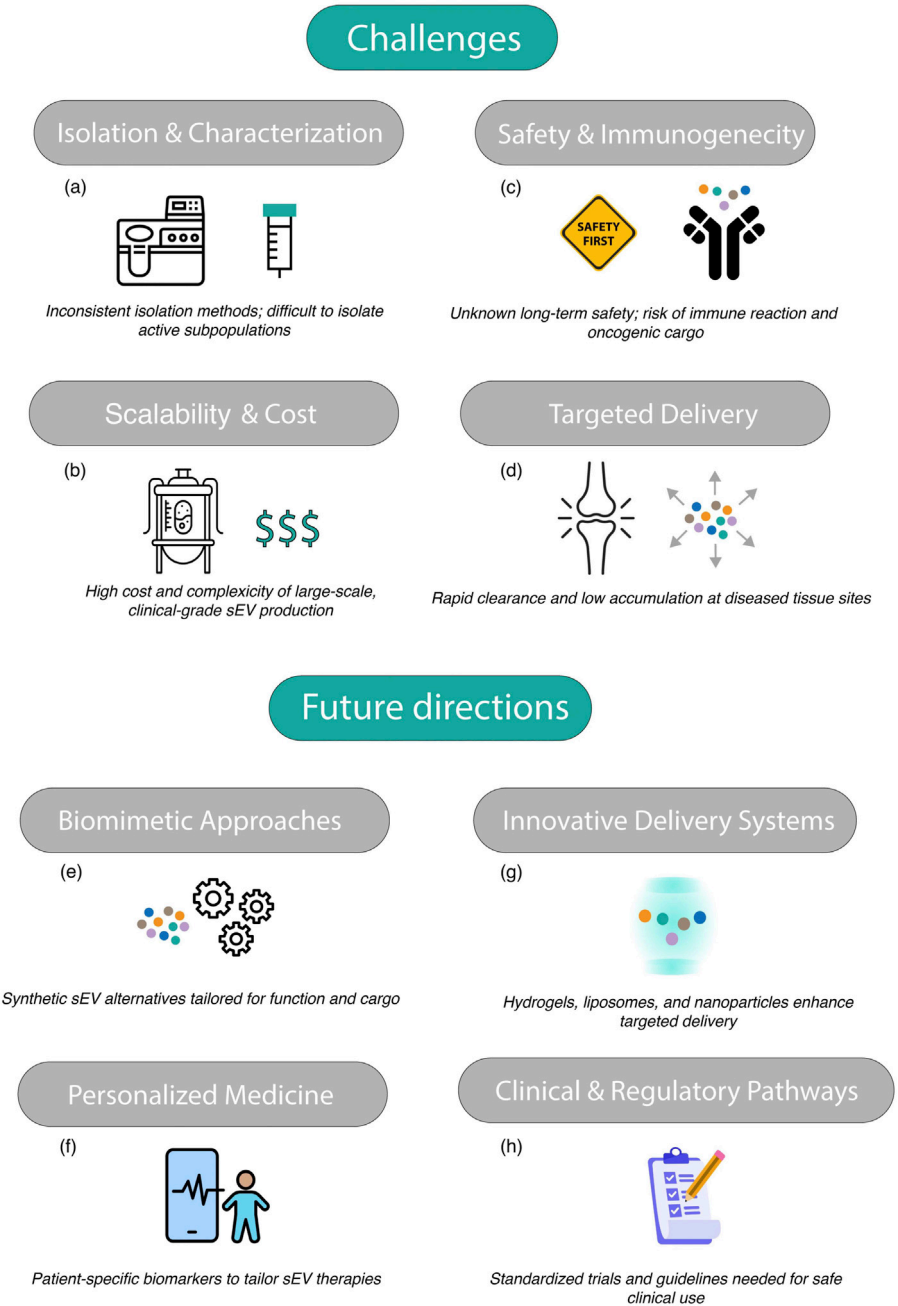
Graphical abstract of challenges and future directions of targeting OA with sEV. *Challenges:* **(a)** isolation and characterization, **(b)** scalability and cost, **(c)** safety and immunogenecity, **(d)** targeted delivery; *Future directions:* **(e)** Biomimetic approaches, **(f)** personalized medicine, **(g)** innovative delivery systems, **(h)** clinical and regulatory pathways.

The standardization of sEV isolation and characterization techniques is a critical challenge. Existing methods such as ultracentrifugation, size-exclusion chromatography, and polymer-based precipitation vary widely in their yield, purity, and reproducibility (Hayami et al., 2006; Otahal et al., 2020; Mustonen et al., 2021; Sanjurjo-Rodríguez et al., 2021; Filali et al., 2022; Zhang et al., 2023). The heterogeneity of sEV populations further complicates their characterization, making it difficult to identify the most therapeutically active subpopulations (Figure 5a). Reliable and scalable methods for isolating specific sEV subsets are urgently needed to ensure consistency in clinical grade sEV preparations.

#### 5.1.2 Scalability and cost

Producing sEV on a large scale while maintaining their functional integrity is a significant barrier to their widespread adoption (Wang et al., 2020; Duan et al., 2021; Liu W. et al., 2023; Warmink et al., 2023; Asghar et al., 2024; Mustonen et al., 2024; Yan et al., 2024). The culture systems required for generating clinical-grade sEV are resource-intensive, and the downstream purification processes are time-consuming and costly (Figure 5b). For example, the use of bioreactors for large-scale sEV production can introduce additional complexity and variability. Achieving cost-effective scalability is critical for translating preclinical successes into accessible clinical therapies.

#### 5.1.3 Safety and immunogenicity

While sEV are generally considered biocompatible, their long-term safety profiles in human applications remain unclear (Gupta et al., 2021; Ragni et al., 2022b; Kouroupis et al., 2023; Ossendorff et al., 2023). Concerns include potential off-target effects, such as unintended interactions with non-target tissues, and the risk of eliciting immune responses (Figure 5c). Additionally, the possibility of transferring pathogenic molecules, such as oncogenic miRNAs or viral particles, underscores the need for rigorous quality control and safety testing.

#### 5.1.4 Delivery to target tissues

Efficiently delivering sEV to specific tissues, such as OA joints, remains challenging. After systemic administration, sEV are often rapidly cleared by the reticuloendothelial system, reducing their bioavailability at target sites (Fosang et al., 2000; Hayami et al., 2006; Otahal et al., 2020; Mustonen et al., 2021; Sanjurjo-Rodríguez et al., 2021; Xu et al., 2021; Filali et al., 2022; Wang et al., 2022; Zhang et al., 2023; Ohtsuki et al., 2024) (Figure 5d). Strategies to enhance the stability and retention of sEV *in vivo* are required to improve their therapeutic efficacy.

### 5.2 Future directions

#### 5.2.1 Biomimetic approaches

One promising strategy to address scalability and production challenges is the development of biomimetic alternatives, such as artificial sEV or synthetic nanoparticles designed to replicate the functional properties of natural sEV (Wu et al., 2022; Jing et al., 2024; Kim et al., 2024; Yan et al., 2024) (Figure 5e). These engineered vesicles can be tailored to carry specific cargos, such as chondroprotective molecules or anti-inflammatory factors, while avoiding the complexities associated with isolating natural sEV.

#### 5.2.2 Personalized medicine

Advances in omics technologies are paving the way for personalized sEV-based therapies. By identifying patient-specific biomarkers, it may be possible to design tailored sEV treatments that address the unique molecular and cellular characteristics of each patient’s condition (Madsen et al., 2010; Matejova et al., 2023; Wu X. et al., 2024) (Figure 5f). For example, sEV could be engineered to carry specific miRNAs or proteins that counteract disease-specific pathways, enhancing their therapeutic efficacy.

#### 5.2.3 Innovative delivery systems

To improve the targeted delivery and bioavailability of sEV, innovative delivery systems such as hydrogels (Zhou et al., 2024), liposomes, or nanoparticle-based carriers are being explored (Hu et al., 2020; Kim et al., 2024). For instance, hydrogels can provide sustained release of sEV within osteoarthritic joints, enhancing their therapeutic effects (Figure 5g). Nanoparticle systems can also be functionalized with surface ligands to target specific cell types, further improving delivery precision.

#### 5.2.4 Clinical trials and regulatory frameworks

The successful translation of sEV therapies into clinical practice requires robust clinical trials to establish their safety and efficacy (Figueroa-Valdés et al., 2025). Large-scale, randomized trials with standardized protocols are needed to generate high-quality evidence (Figure 5h). Additionally, the development of clear regulatory guidelines for the production, characterization, and clinical use of sEV will be critical for their widespread adoption.

## 6 Conclusion

sEV have emerged as a transformative tool in regenerative medicine, offering a multifaceted approach to addressing the challenges of OA and other degenerative diseases. By delivering bioactive molecules that promote chondrogenesis, modulate inflammation, and stimulate angiogenesis and tissue repair, sEV hold great promise for advancing the field of therapeutic interventions.

Despite these advancements, significant challenges remain on the path to clinical translation. Standardizing isolation techniques, achieving scalable production, ensuring safety, and developing efficient delivery systems are essential steps toward realizing the full therapeutic potential of sEV. Biomimetic technologies, personalized medicine, and advanced delivery strategies represent exciting avenues for overcoming these hurdles.

Looking forward, rigorous clinical trials and collaborative efforts among researchers, clinicians, and industry stakeholders will be instrumental in establishing sEV therapies as a cornerstone of modern medicine. As research continues to evolve, sEV are poised to revolutionize the treatment of osteoarthritis and other chronic conditions, offering hope for improved patient outcomes and quality of life.

## Data Availability

The raw data supporting the conclusions of this article will be made available by the authors, without undue reservation.

## Glossary

3′-UTR: 3′untranslated region
ACLT: Anterior Cruciate Ligament Transection
AD-MSCs: Mesenchymal Stem Cells from Adipose Tissue
ADAMTS-4: DAM metallopeptidase with thrombospondin type 1 motif 4
ADAMTS-5: DAM metallopeptidase with thrombospondin type 1 motif 5
ALK3: Bone morphogenetic protein receptor type-1A
AM-MSCs: Mesenchymal Stem Cells from Ammiotic Membrane
APOM: Apolipoprotein M
ARRIVE: Animal Research: Reporting of *in vivo* Experiments
AVV: Adeno-Associated Virus
BM-MSCs: Mesenchymal Stem Cells from Bone Marrow
BMP-2: Bone Morphogenetic Protein 2
BMPs: Bone Morphogenetic Proteins
C3: General transcription factor 3C polypeptide 3
C4B: C4b-binding protein β chain
CD10: Neprilysin
CD326: Epithelial cell adhesion molecule
CD45: Receptor-type tyrosine-protein phosphatase C
CD56: Neural cell adhesion molecule 1
CH: Chondrocytes
CIOA: Collagenase-Induced OA
circRNAs: Circular RNAs
COL2A: Collagen α-1(II) chain
CPEB1: Cytoplasmic polyadenylation element-binding protein 1
DM4: Glyceraldehyde-3-phosphate dehydrogenase
DMM: Destabilization of the meniscus
DP-SCs: Dental Pulp-derived Stem Cells
DPYSL2: Dihydropyrimidinase-related protein 2
E2F2: Transcription factor E2F2
ECM: Extracellular Matrix
ENPP1: Ectonucleotide pyrophosphatase/phosphodiesterase family member 1
ERK: Mitogen-activated protein kinase 4
ESCs: Embryponic Stem Cells
EV: Extracellular Vesicle
FGF-2: Fibroblast Growth Factor-2
Ha: Hyaluronic Acid
HGF: Hepatocyte Growth Factor
IGF-1: Insulin-like Growth Factor-1
IGF1R: Insulin-like growth factor 1 receptor
IL-1: Interleukin-1
IL-10: Interleukin-10
IL-13: Interleukin-13
IL-17: Interleukin-17
IL-1β: Interleukin-1β
IL-35: Interleukin-35
IL-4: Interleukin-4
iNOS: Nitric oxide synthase, inducible
IPFP/IFP-MSCs: Infrapatellar Fat Pad-derived Mesenchymal Stem Cells
iPSC-derived MSCs: Mesenchymal Stem Cell-derived iPSCs
iPSCs: induced Pluripotent Stem Cells
iTRAQ: Isobaric Tag for Relative and Absolute Quantitation
JAK/STAT: Tyrosine-protein kinase JAK/Signal transducer and transcription activator
JAK2/STAT3: Tyrosine-protein kinase JAK2/Signal transducer and transcription activator-3
KL: Kellgren Lawrence
lncRNA: long non-coding RNAs
MAPK: MAP kinase-activated protein kinase 3
MDM2: E3 ubiquitin-protein ligase Mdm2
METTL3: N(6)-adenosine-methyltransferase catalytic subunit METTL3
MFG-E8: Lactadherin
MIA: Monosodium Iodoacetate
miRNAs/miR: microRNAs
MMP-13: collagenase-3
MMP-3: stromelysin-1
MMPs: matrix metalloproteinases
MMx: Resection of Medial Menisci
MSCs: Mesenchumal Stem Cells
mTOR: Serine/threonine-protein kinase mTOR
MYH9: Myosin-9
ncRNAs: noncoding RNAs
NEAT1: Protein HEXIM1
NF-κB: Nuclear factor NF-kappa-B
NLRP3: NACHT, LRR and PYD domains-containing protein 3
Nrf2: Nuclear factor erythroid 2-related factor 2
OA: Osteoarthritis
OSM: Oncostatin M
p53: Cellular tumor antigen p53
p75NTR: p75 Neurotrophin Receptor
PESCs: Progenitor Endothelial Stem Cells
PHLDA2: Pleckstrin homology-like domain family A member 2
PI3K/Akt: Phosphatidylinositol 3-kinase catalytic subunit type 3/RAC-β serine/threonine-protein kinase
PKC: Protein kinase C α type
PPD-MSCs: Positively charged MSC-sEV
PRISMA: Preferred Reporting Items for Systematic Reviews and Meta-Analyses
proNGF: pro-β -nerve growth factor
RA: Rheumatic Arthritis
S-MSCs: Mesenchymal Stem Cells from Synovial membrane
S100A8: Protein S100-A8
sEV: small Extracellular Vesicles
SF: Synovial Fluid
SFRP5: Secreted Frizzled-Related Protein 5
SJ-cells: Cells from Synovial Joint
SOD3: Extracellular superoxide dismutase [Cu-Zn]
SOX11/FOXO: Transcription factor SOX-11/Forkhead box protein O
SOX9: Transcription factor SOX-9
STAT3: Signal transducer and transcription activator-3
T-MSCs: Tonsil-derived Mesenchymal Stem Cells
TGF-β: Transforming Growth Factor-β
TGF-β3: Transforming Growth Factor-β3
TIMP-3: Tissue Inhibitors of Metalloproteinases-3
TIMPs: Tissue Inhibitors of Metalloproteinases
TMJOA: Temporomandibular Joint Osteoarthritis
TNF-α: Tumor Necrosis Factor-α
Trim14: Tripartite motif-containing protein 14
Trx80: Recombinant human Thioredoxin 80
TSG-6: Tumor necrosis factor-inducible gene 6 protein
UAC: Anterior Crossbite Models
UC-MSC: Mesenchymal Stem Cells from Umbilical Cord
VEGF: Vascular Endothelial Growth Factor
Wnt: Protein Wnt
YAP: Transcriptional coactivator YAP1
εPL-PEG-DSPE: ε-polylysine-polyethylene-distearyl phosphatidylethanolamine
